# Neglected Biodiversity of Fish Assemblages Associated With Antipatharia (Black Corals) on Tropical Shallow Reef Ecosystems

**DOI:** 10.1002/ece3.72015

**Published:** 2025-08-21

**Authors:** Erika Gress, Kevin R. Bairos‐Novak, Tom C. Bridge, Gemma F. Galbraith

**Affiliations:** ^1^ ARC Centre of Excellence for Coral Reef Studies James Cook University Townsville Queensland Australia; ^2^ College of Science and Engineering James Cook University Townsville Queensland Australia; ^3^ Biodiversity and Geosciences Program, Museum of Tropical Queensland Queensland Museum Network Townsville Queensland Australia; ^4^ AIMS@JCU Australian Insitute of Marine Science Townsville Queensland Australia

**Keywords:** Great Barrier Reef, habitat provision, Orpheus Island, reef management and conservation, SS Yongala

## Abstract

Addressing anthropogenic threats compromising the persistence of tropical marine ecosystems requires a comprehensive understanding of the fundamental ecological functions organisms fulfill in these realms. Habitat provision is a paramount function of corals in tropical marine ecosystems, although most research in this area has concentrated on scleractinians (hard corals). Here, we provide one of the first empirical studies of fish communities on shallow tropical reefs associated with another, lesser‐known hexacoral group—the antipatharians (black corals). We quantify (i) the abundance, and taxonomic and functional diversity of fish communities associated with antipatharians and (ii) the type of associations between the fish and the antipatharian colonies. Surveys were conducted on an artificial reef (*SS Yongala* shipwreck) and on a coral reef (Orpheus Island) in the central Great Barrier Reef, Australia. We documented 28 different species of fish within seven trophic groups and 23 functional entities associated with antipatharians, predominantly using the colonies as shelter. Antipatharians support both taxonomically distinct fish assemblages (> 40% of species) and unique types of associations with the fishes compared to scleractinians. At the functional level, we observed a large overlap in the fish community between antipatharians and scleractinians, reflecting their shared ecological roles, although antipatharians support significantly higher functional diversity. Given the similarity in functional composition of fish assemblages utilising both antipatharians and scleractinians, the presence of antipatharians may help buffer the effects of ongoing hard coral decline in tropical marine ecosystems. Overall, our study provides empirical evidence of the important role of antipatharians in supporting fish functional and taxonomic diversity on shallow tropical reefs.

## Graphical abstract

The role of antipatharians (black corals) in supporting tropical shallow reef fishes.

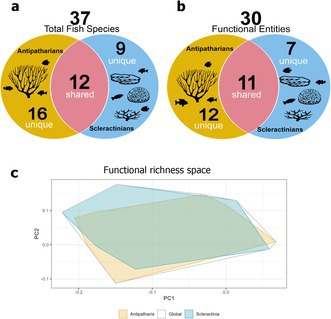

## 1 Introduction

Identifying, understanding and maintaining ecological functions is essential to sustaining ecosystems in the face of current anthropogenic stressors (Bellwood et al. 2004; Brandl, Rasher, et al. 2019; Hughes et al. 2017). Traditionally, ecological studies have focused on the taxonomic composition of assemblages. However, there has been an increasing focus on understanding the functional roles of species in recognition of the fact that relatively few taxa perform key ecological functions (Bellwood et al. 2004; Harborne et al. 2017; Naeem et al. 2012). Although species richness plays an important role in buffering reef ecosystem functions (Lefcheck et al. 2021), the presence and abundance of functionally important species are particularly important for ecosystem resilience (McGill et al. 2006). For example, in coral reef ecosystems, a global analysis of unique trait combinations of fish showed that, in the Central Indo‐Pacific, about one‐third of ecological functions are provided by only one species (Mouillot et al. 2014).

Structural complexity is one of the most important ecological traits on reefs and has been associated with key coral taxa (Darling et al. 2017; González‐Barrios and Álvarez‐Filip 2018; Kerry and Bellwood 2015a). Three‐dimensional habitat strongly influences the composition and diversity of a range of reef‐associated taxa, particularly fishes (Jones and Syms 1998; Wilson et al. 2009). Reef complexity has been shown to influence species richness, density, biomass and trophic structure of fish assemblages (Behrents 1987; Beukers et al. 1997; Darling et al. 2017; Gratwicke and Speight 2005). The abundance and size of holes or cavities mediate predation dynamics and juvenile fish survivorship, thereby influencing the composition of fish communities in different ways (Almany 2004; Darling et al. 2017; Lingo and Szedlmayer 2006). Habitat complexity on coral reefs can be provided by both the underlying reef substrate and by habitat‐forming sessile benthos such as corals, algae and sponges. The loss of habitat‐forming benthos and resulting loss of habitat complexity therefore compromises the ecological functioning of coral reefs (Graham and Nash 2013) and makes them less likely to recover from disturbances (Graham et al. 2015).

Most studies examining the importance of habitat‐forming benthos on coral reefs have focused on scleractinians (hard corals). For example, the abundance of scleractinians with complex growth forms (e.g., *Acropora* and *Pocillopora*) is often correlated with the composition of fish communities on shallow reefs (< 30 m depth) (Beukers et al. 1997; Darling et al. 2012; Kerry and Bellwood 2015a). Similarly, a study in the Great Barrier Reef (GBR) showed that tabular *Acropora* spp. had disproportionate effects on the distribution of large reef fish communities, even when that morphology constituted a small fraction (4%) of the total benthic cover (Kerry and Bellwood 2015a). Moreover, a branching morphology can provide fine‐scale structural complexity for small‐bodied and/or juvenile fishes to refuge from predators (Beukers et al. 1997). Despite the importance of coral morphology and size (Fisher 2023; Zawada et al. 2019), most coral reef monitoring programs only document live coral cover, without considering structural complexity.

Structural complexity at reefscape scales can be estimated by visual scores (Gratwicke and Speight 2005), although these approaches can be easily influenced by surveyor perspectives. More recently, photogrammetry has enabled quantitative analysis of reef structural complexity (Ferrari et al. 2016; Friedman et al. 2012; Kornder et al. 2021). Photogrammetry has been used to quantify the total volume of shelter (habitat) provided by different scleractinian growth forms or ‘shelter volumes’, and predictive models of shelter volume (a 3D metric) can be estimated based on 2D metrics (area or diameter) for each major growth form (Aston et al. 2022; Urbina‐Barreto et al. 2021). Therefore, it is currently possible to quantify the shelter volume of different coral morphologies and investigate the link between coral complexity and reef fish abundance at finer spatial scales (Urbina‐Barreto et al. 2022). To date, this research has focused almost entirely on scleractinians, with no attempts to quantify the importance of other habitat‐forming benthic groups in providing habitat complexity.

Antipatharians—commonly known as black corals—are a sister group to the scleractinians, within the class Hexacorallia. Antipatharians occur in all worlds' oceans except for the Arctic, at depths ranging from 1 to 8900 m (Molodtsova et al. 2008; Pasternak 1977; Wagner et al. 2012). Unlike scleractinians, antipatharians do not produce a calcium carbonate skeleton but a thorny axial skeleton (brown or black in colouration) composed of different scleroproteins (Goldberg 1978; Goldberg et al. 1994). Antipatharians have a range of morphologies including flabellate (fan‐like), whip‐like, bottle‐brush‐like and branching (which can be either small bush‐like or large arborescent colonies) (Wagner et al. 2012). Despite limited studies on the topic, it is known that antipatharians provide important habitat complexity supporting an array of fish. For example, (Boland and Parrish 2005) examined the diversity and movement patterns of fish associated with branching antipatharians between 52 and 73 m depth in Hawaii. Although their study was conducted in a mesophotic coral ecosystem (MCEs; 30–150 m depth reefs), 95% of the fish recorded also occur on shallow reefs (Boland and Parrish 2005). A study on the subtropical eastern Atlantic on antipatharian forests (i.e., dense aggregations of branching antipatharian colonies) found that 90% of fish functional richness inhabiting antipatharian forests at mesophotic depths were also found on shallow reefs, although the dominant species varied between shallow and mesophotic depths (Bosch et al. 2023). In temperate mesophotic ecosystems (TMEs) in the Mediterranean Sea, an array of fishes—including species of both conservation interest and high commercial value—were associated with antipatharian forests (Chimienti et al. 2022).

Despite the clear importance of antipatharians as habitat for a wide range of fish species across a range of ecosystems, there is currently little information on their role on shallow tropical reefs. Moreover, no studies have examined whether antipatharians host a different fish community from the one in association with neighbouring scleractinian corals. Here, we provide the first assessment of the fish community structure associated with antipatharians in shallow reef ecosystems and explore how this previously overlooked benthic taxon influences fish communities on these reefs. We quantified the fish species richness and density, and recorded the behaviour of fishes in close association with both antipatharian and scleractinian colonies at two sites in the central Great Barrier Reef (GBR) to investigate: (i) the fish community structure associated with antipatharians and (ii) the effects of coral taxon, corals complexity properties (area and shelter volume) and reef sites on the fish communities. This information aims to improve our understanding of the role of antipatharians in supporting functional and taxonomic diversity on reefs.

## 2 Methods

### 2.1 Field Sites

Multiple in situ surveys were conducted at two locations on the central Great Barrier Reef, Queensland, Australia, between May and October 2021: the *SS Yongala* wreck and Orpheus Island. The *Yongala* is a 107 m long wreck located ~22 km from the mainland (−19.291, 147.627) and sits between 14 and 29 m depth. The wreck is a world‐renowned dive site for its high fish abundance; however, with the exception of one study of the fish species richness conducted in the late 1990s (Malcolm et al. 1999), scientific studies of the abundance and diversity of fish and benthic fauna are lacking. The wreck supports both antipatharian and scleractinian corals and therefore represents a great opportunity to investigate the influence of both coral taxa on fish communities. Because the *Yongala* is in essence an ‘artificial’ reef, and to explore the generality of our results across shallow coral reefs, we also collected data on a well‐studied shallow reef that, like the *Yongala*, supports interspersed populations of antipatharians and scleractinians between 13 m and 16 m depth—Orpheus Island (18.616, 146.519, at Little Pioneer and Iris Point). The *Yongala* and Orpheus Island are ~142 km apart (Map in Appendix S1a). Both sites are within No‐Take Marine Protected Areas (Marine Park zones), and the *Yongala* is a Commonwealth Cultural Heritage Site.

### 2.2 Corals Area and Shelter Volume

Photos with a scale were taken to estimate the planar area of each of the coral colonies using the software ImageJ (Bourne 2010). For scleractinians, we recorded the planar area as viewed from above, which is the traditional approach (Rogers et al. 1994) and also the method used by Urbina‐Barreto et al. (2021) to develop predictive models of shelter volume. For antipatharians, planar area was calculated based on width (diameter) and height of the colonies as viewed from the side, which are considered the best estimators of surface area for non‐scleractinian branching bushy‐like coral colonies (Santavy et al. 2013). The shelter volume (dm^3^) of all coral colonies was calculated using the predictive models (based on the colonies diameter) of Urbina‐Barreto et al. (2021) for branching, massive and tabular colonies. No predictive models are available for encrusting and foliose growth forms; thus, these two morphologies were treated as massive. Predictions of shelter volume were made using log‐scale colony diameters, which were nearly identical to the predicted shelter volumes when using area (Shelter volume figures and images of coral morphologies are presented in Appendix S1b).

### 2.3 Fish Surveys

Four‐minute long stationary videos of both antipatharian and scleractinian colonies were filmed at 30 fps on SCUBA during daylight hours (1100–1400). Coral colonies were stochastically (i.e., as encountered on the dives) filmed in pairs (one for each coral taxon)—where the antipatharian and scleractinian were at the same depth, ≤ 10 m apart, and filmed at the same time or one immediately after the other. At *Yongala*, 17 coral pair videos were filmed (34 colonies) at two depth ranges: 14–20 and 21–27 m (eight and nine colony pairs, respectively). At Orpheus Island, scleractinians are not abundant beyond 14 m depth; therefore, six coral pair videos (12 colonies) were filmed at 14 m depth, where both coral taxa coexist, and to maintain a similar depth range to the other site.

### 2.4 Video Analysis

We identified observed fish communities to species and recorded the maximum number of fish individuals of a particular species that occurred in a single frame of a video (MaxN) using EventMeasure (SeaGIS, Melbourne Australia), which allowed us to estimate the abundance of each fish species observed. This represents a conservative estimate of the minimum number of individuals known to have been present in the sampling area (each colony) over the filming period and provides the maximum number of individuals of a particular species occurring in a single frame of a video (Lyle et al. 2007). We also recorded the ‘behaviour’ of each fish associating with coral colonies as follows:

- *HovA*—hovering around (< 50 cm around the colonies)
- *HovH*—hovering around and hiding (hovering < 50 cm around the colonies and seeking refuge among the coral structure)
- *Stat_next*—static (resting next to the coral colonies)
- *Stat_in*—static (static within or on the coral colonies)
- *Feed*—feeding on polyps (in the case of corallivores), or feeding on algae on top of coral (e.g., Scarus)
- *Clean*—being cleaned (by *Labroides dimidiatus* )
- *Pass—*passing by

Filming of footage initialised immediately after encountering the colonies, and for video analysis fish were not counted for the first 10 s. This approach maximises the data being collected—considering we had time constraints of working at deeper depths on *Yongala*—while optimising the likelihood of detecting both cryptic and mobile species that may not be present if longer wait times were used (Bohnsack and Bannerot 1986; Willis 2001). This approach was standardised across all videos (filming and analyses). Fishes passing by (*Pass*) were recorded; however, these were not considered for further analysis because of the uncertainty of their association with the corals. For instance, fish passing could have been foraging but were not observed consuming their prey.

### 2.5 Statistical Analysis

All analyses were conducted in R 4.4.0 (R Core Team 2024). The shelter volume (dm^3^) of all coral colonies was calculated using the predictive models proposed by Urbina‐Barreto et al. (2021) as described above. To standardise fish abundance and species richness, we used the area of the surveyed coral colonies (i.e., we used fish density m^−2^ and fish richness m^−2^).

To explore the effect of depth, we fit generalised linear mixed‐effects models (GLMMs) examining the effect of the two depth bands at *Yongala* (14–20 and 22–27 m) and coral order (Antipatharia and Scleractinia) on fish density m^−2^ and species richness m^−2^. GLMMs were fit using restricted maximum likelihood with a gamma distribution (log‐link) and a random effect of colony pair ID. For each model, we included the effect of coral order and fit models using a full interaction term. Where interactions were significant, we examined pairwise contrasts and split the analysis by coral order for subanalyses using generalised linear models (no pair ID effect). The effect of depth on fish species richness m^−2^ for scleractinians did not meet the assumption of homogeneity of variance; therefore, we fit a generalised least squares (GLS) model with the variance function ‘varIdent’ to account for heteroscedasticity across groups (i.e., unequal variances of species richness m^−2^ at each depth band). To investigate the effect of site and coral taxa on fish density m^−2^ and species richness m^−2^, we again fit gamma log‐link GLMMs using colony pair as a random effect. Additionally, for both depth and site models, we created AICc model selection tables that confirmed full‐factorial model results (see Appendix S2).

To quantify reef fish functional diversity supported by antipatharian and scleractinian colonies, we constructed a multidimensional trait space using the R package mFD (v1.1.2; Magneville et al. 2022). We selected six ecologically relevant traits reflecting key aspects of fish functional roles on coral reefs: fish body size (0–7, 7.1–15, 15.1–30, 30.1–50, 50.1–80 and > 80 cm), diet category (herbivore‐detritivores, macroalgae‐herbivores, sessile invertivores, mobile invertivores, planktivorous, piscivores and omnivores), mobility (sedentary, mobile within a reef or mobile between reefs), activity period (diurnal, diurnal and nocturnal or nocturnal), group size (solitary, pairing, small groups of 3–20 individuals, medium groups of 20–50 individuals, or large groups of > 50 individuals) and vertical position in the water column (benthic, bentho‐pelagic or pelagic). Fish traits were chosen following the framework developed by Mouillot et al. (2014) and compiled from FishBase (Froese and Pauly 2024) and supplemented with values from primary literature where database entries were missing or ambiguous. We calculated pairwise Gower distances between functional entities, which accommodate mixed trait types and allocate equal weighting (Legendre and Legendre 2012). A Principal Coordinates Analysis (PCoA) was then applied to the distance matrix using the first four principal axes. The number of axes to construct functional trait space was chosen based on mean absolute deviation (msD) based on the deviation between initial trait‐based distances and distances in the functional space (Maire et al. 2015). To visualise this analysis, we plotted convex hulls of functional trait space from fish assemblages on antipatharians, scleractinians and the global trait space (all colonies combined) using ggplot (Wickham 2016). Data, analyses, and a summary table of fish functional analyses are available in Appendix S3. Additionally, fish functional diversity indices were estimated as follows: (a) a GLMM was fit using a Gaussian distribution with site as a random effect to assess mean functional entities (i.e., unique combinations of functional traits); and (b) functional redundancy (i.e., multiple species performing same or similar ecological roles) was estimated using a Generalised Additive Models for Location, Scale and Shape (GAMLSS) with a gamma distribution to account for under‐dispersed and skewed data.

All GLMMs were fit using restricted maximum likelihood (REML) via the package *glmmTMB* (Brooks 2022) and model diagnostics (i.e., assumptions of normality, homogeneity or variances, no overdispersion) were assessed using the package *DHARMa* (Harting 2022). Post hoc analysis, estimated marginal means and pair‐wise contrasts were done using the package *emmeans* (Lenth et al. 2022); and predicted values were calculated using the *predict* function with 95% confidence intervals. The GAMLSS model was fit using the *gamlss* package (Stasinopoulos and Rigby 2007) and residuals were examined using the function *residuals* in the same package. All models' formulas, results and summary statistics—marginal means and 95% confidence intervals—are reported in Appendix S2.

To visualise the overall significance of the different variables (site, coral taxa, shelter volume) on explaining the fish community structure, a constrained ordination using distance‐based redundancy analysis (dbRDA; Legendre and Andersson 1999) was conducted using the package *vegan* (Oksanen 2022), with the variables overlaid as a vector. This was followed by a permutation‐based multivariate ANOVA (PERMANOVA) of the dbRDA to identify significant (*p* < 0.05) variables driving the fish community structure. Then, a one‐way permutation‐based multivariate ANOVA (PERMANOVA) was performed using the function ‘adonis2’ in vegan to further assess findings. To validate our interpretation on the PERMANOVA, we performed a PERMDIST test *betadisp* (a multivariate equivalent to Levene's test for homogeneity of variance); test results are available in Appendix S2.

## 3 Results

### 3.1 Effect of Depth on Fish Density m^−2^ and Species Richness m^−2^ at Yongala

We found no significant (*p* > 0.05) differences between depths for either fish density m^−2^ or species richness m^−2^ (Table 1; Appendix S2). Mean fish density m^−2^ for antipatharians in the shallower depth range (14–20 m) was 2.23 ± 0.93, and 0.65 ± 0.32 for scleractinians. In the deeper depth range (22–27 m), antipatharians hosted an average fish density m^−2^ of 1.99 ± 0.80 and scleractinians hosted fish densities of 0.58 ± 0.24. Similar to fish density, species richness did not strongly vary across depth bands. Mean species richness m^−2^ for antipatharians in the shallower depth range (14–20 m) was 0.089 ± 0.024, and 0.052 ± 0.014 for scleractinians. In the deeper depth range (22–27 m), antipatharians hosted an average fish density m^−2^ of 0.054 ± 0.014 and scleractinians hosted fish densities of 0.124 ± 0.032. Despite having slightly higher richness, scleractinians at the deeper *Yongala* reef band (22–27 m) were not significantly different upon examining pairwise differences in a post hoc analysis (*p* > 0.05; see Appendix S2). Since depth range had no overall effect at *Yongala*, we pooled the fish density m^−2^ and species richness m^−2^ across the two depth bands for subsequent analyses, examining corals in terms of site (*Yongala* vs. Orpheus) and coral order (Antipatharia vs. Scleractinia).

**TABLE 1:** Summary table of the generalised linear mixed‐effect models (GLMMs), generalised linear models (GLMs), generalised least square model (GLS) and Generalised Additive Models for Location, Scale and Shape (GAMLSS) model.

Model	Question	Formula [model type]	Parameters	Test statistic	*p*
Model 1	Depth on fish density m^−2^, for all colony pairs at *Yongala* site	Density m^−2^ ~ Depth range × Coral taxon + (1|Pair) [GLMM]	Intercept (14–20 m, Antipatharia)	1.91	0.057
			Depth range effect (22–27 m)	−0.19	0.85
			**Coral taxon effect (Scleractinia)**	**−2.15**	**0.032**
			Depth × coral taxon interaction	0.00	0.99
Model 2	Depth on fish richness m^−2^, for all colony pairs at *Yongala* site (two depth bands)	Richness m^−2^ ~ Depth range × Coral taxon + (1|Pair) [GLMM]	Intercept (14–20 m, Antipatharia)	−8.99	< 0.001
			Depth range effect (22–27 m)	−1.34	0.18
			Coral taxon effect (Scleractinia)	−1.39	0.17
			**Depth × coral taxon interaction** (Note: all confidence intervals overlap and no significant pairwise differences found during post hoc analysis)	**2.59**	**0.01**
Model 2a	Depth on fish richness m^−2^, for antipatharians at *Yongala*	Richness m^−2^ ~ Depth range [GLM]	Intercept (14–20 m)	−11.6	< 0.001
			Depth range effect (22–27 m)	−1.73	0.083
Model 2b	Depth on fish richness m^−2^, for scleractinians at *Yongala*	Richness m^−2^ ~ Depth range [GLS]	Intercept (14–20 m)	−13.5	< 0.001
			Depth range effect (22–27 m)	0.90	0.38
Model 3	Coral taxon and site on fish density m^−2^	Density m^−2^ ~ Coral taxon * Site + (1 | Pair) [GLMM]	Intercept (Antipatharia, Orpheus)	−2.48	0.013
			**Coral taxon effect (Scleractinia)**	**−2.33**	**0.020**
			**Site effect (Yongala)**	**3.66**	**< 0.001**
			Coral taxon × Site interaction	0.16	0.87
Model 4	Coral taxon and site on fish richness m^−2^	Richness m^−2^ ~ Coral taxon * Site + (1 | Pair) [GLMM]	Intercept (Antipatharia, Orpheus)	−12.1	< 0.001
			Coral taxon effect (Scleractinia)	−1.10	0.27
			**Site effect (Yongala)**	**2.87**	**0.0041**
			Coral taxon × site interaction	1.44	0.15
Model 4	Coral taxon on mean fish entities	Functional entities ~ Coral taxon + (1|SITE) [GLMM]	Intercept (Antipatharia)	4.30	< 0.001
			**Coral taxa effect (Scleractinia)**	**2.87**	**0.0064**
Model 6	Coral taxon on mean fish functional redundancy	Functional redundancy ~ Coral taxon [GAMLSS]	Intercept (Antipatharia)	4.30	< 0.001
			Coral taxon effect (Scleractinia)	−1.10	0.276

*Note:* Model numbers (first column) correspond to the name given on Appendix S2, which contains full model summaries, estimated marginal means and contrast analysis results. Significant factors in each model are in bold. *Density* m^−2^ refers to the standardised fish abundance per m^2^; and *Richness* m^−2^ refers to the number of fish species observed per m^2^.

### 3.2 Effect of Coral Taxon and Site on Fish Density m^−2^ and Species Richness m^−2^

Fish density m^−2^ varied significantly between the coral taxa (Table 1—Model 3), with antipatharians hosting on average 3.55 times the fish density per area compared to scleractinians (Appendix S2). Coral taxon did not have a significant effect on fish richness m^−2^ (Table 1—Model 4; Appendix S2). For antipatharians, fish density m^−2^ decreased from 2.13 ± 0.56 fish m^−2^ at *Yongala*, to 0.35 ± 0.15 fish m^−2^ at Orpheus. Likewise, for scleractinians, fish density decreased from 0.63 ± 0.192 m^−2^ at *Yongala*, to 0.09 ± 0.04 m^−2^ at Orpheus (Figure 2; Appendix S2). At *Yongala*, fish richness m^−2^ for antipatharians was 0.071 ± 0.013 m^−2^, and 0.090 ± 0.016 m^−2^ for scleractinians. At Orpheus, richness m^−2^ was 0.026 ± 0.008 m^−2^ antipatharians, and 0.016 ± 0.005 m^−2^ for scleractinians. Site had a significant effect on fish density and richness per m^2^ (Table 1—Models 3 & 4, respectively), with *Yongala* having 6.44 and 3.93 times the fish density and species richness m^−2^, respectively, compared to Orpheus (Appendix S2).

### 3.3 Fish Community Structure Associated With Antipatharians

**FIGURE 1:**
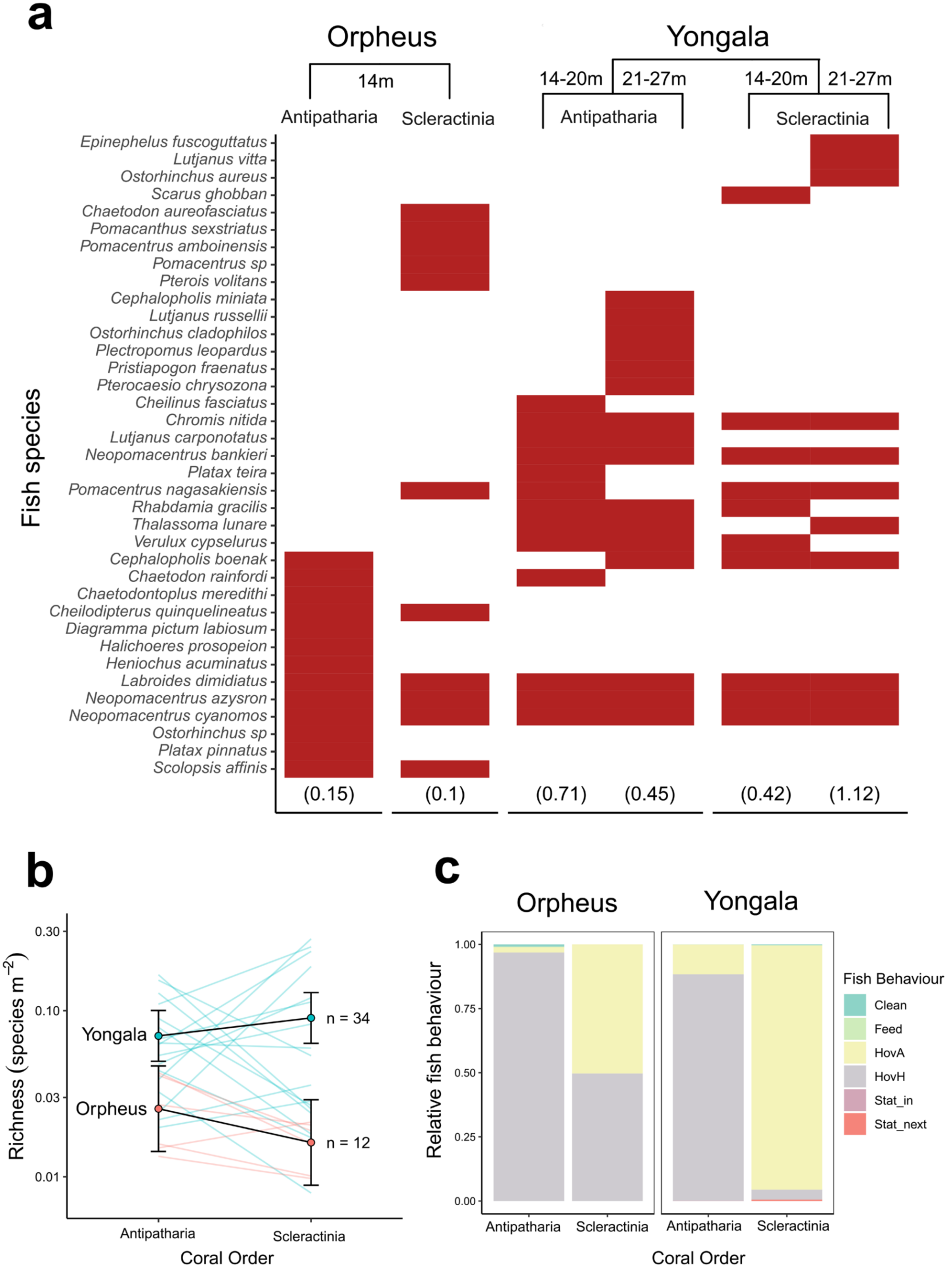
Fish species richness associating with antipatharians and scleractinians at *Yongala* and Orpheus: (a) Fish species present (red bars) or absent (white bars) for each coral taxa, study site and depth; numbers in parenthesis are the total relative fish species richness m^−2^. (b) Marginal effects plot of mean fish richness m^−2^ predicted for each coral taxa (Model 4 in Table 1). Coloured lines connect colony pairs (one antipatharian and one scleractinian) for each of the two sites: 34 colonies at *Yongala* (*n* = 17 colony pairs), and 12 colonies at Orpheus (*n* = 6 colony pairs) surveyed. (c) Relative fish behaviour showing the proportional contribution of each type of behaviour.

**FIGURE 2:**
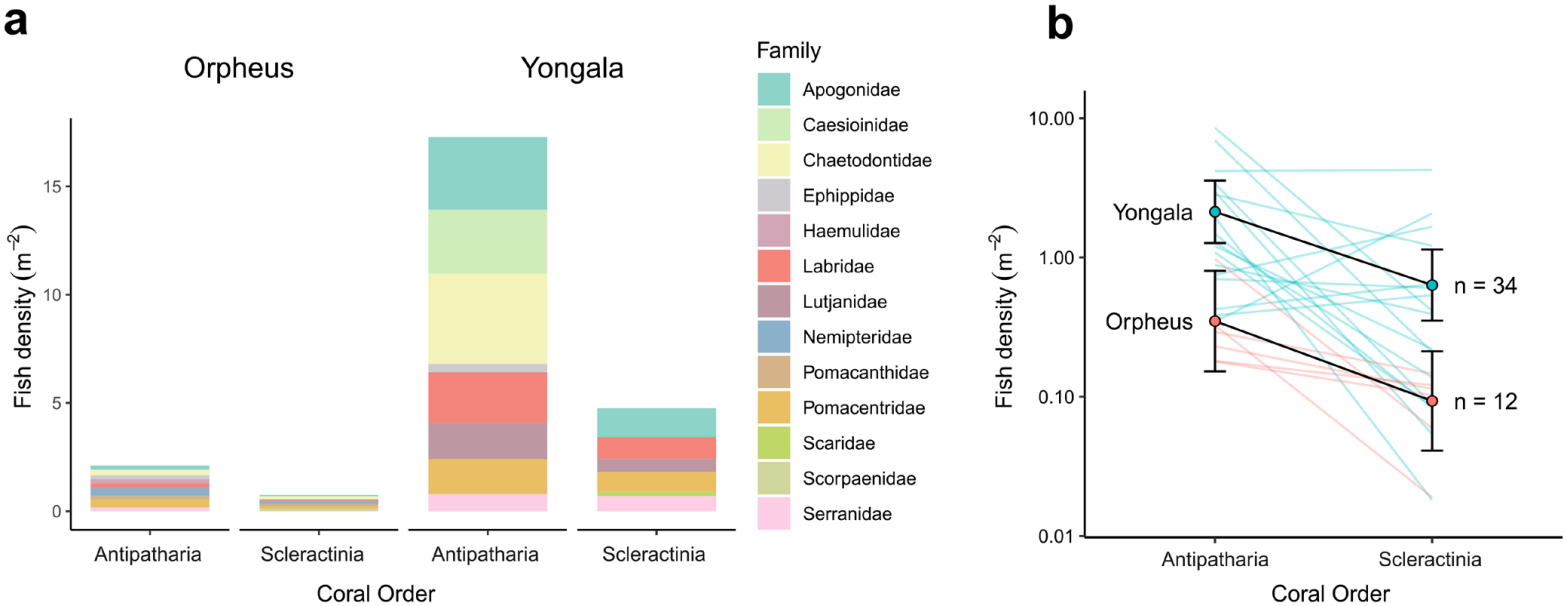
Fish density associating with antipatharians and scleractinians at *Yongala* and Orpheus: (a) Fish density m^−2^ showing the contribution of each family proportional to the average density of fish within the family. (b) Marginal effects plot of mean fish density m^−2^ predicted for each coral taxa (Model 3 in Table 1). Coloured lines connect colony pairs (one antipatharian and one scleractinian) for each of the two sites: 34 colonies at *Yongala* (*n* = 17 colony pairs), and 12 colonies at Orpheus (*n* = 6 colony pairs) surveyed.

A total of 28 fish species were recorded in close association with antipatharians (20 different species at *Yongala* and 13 at Orpheus), from 11 families (Figure 1a; data in Appendix 1), and 23 functional entities (Table 1 in Appendix S3). The most common and abundant species were *Neopomacentrus azysron* , *Rhabdamia gracilis* , *Chromis nitida* , *N. bankieri* , *Verulux cypselurus*, *Ostorhinchus cladophilos*, *Cheilodipterus quinquelineatus* and *N. cyanomos* . The most frequently observed fish behaviours were HovH and HovA, with around 89% and 10% of individuals displaying these behaviours, respectively (Figure 1c). Marginal fish density at *Yongala* was 2.12 ± 0.5 (mean fish density m^−2^ ± SE), and 0.35 ± 0.5 m^−2^ at Orpheus (Figure 2). The fish communities associated with scleractinians in comparison to the antipatharians show the following patterns: they shared > 40% of the species recorded, the most common fish behaviour was HoA (~81% of fish), in contrast to HoH, which was dominant for antipatharians but represented only 18% of the fish associated with scleractinians (Figure 1c). At the functional level, we found that there is an overlap of approximately 37% of functional entities (Table 1 in Appendix S3).

### 3.4 Functional Diversity Indices of Coral‐Associated Fish Assemblages

The overall total number of fish functional entities associated with the corals was 30, of which 12 were exclusively associated with antipatharians and seven uniquely associated with scleractinians (Figure 4b; Appendix S2). The marginal mean estimate of functional entities for antipatharians was 3.9 ± 0.3, and 2.5 ± 0.3 for scleractinians, and it was significantly different between coral taxa (*p* < 0.05; Table 1; Appendix S2). The marginal estimated mean of functional redundancy for antipatharians was 1.16 ± 0.04, and 1.11 ± 0.04 for scleractinians, and it was not significantly different between coral taxa (*p* > 0.05; Table 1; Appendix S2). Functional richness (FRic) was quantified as the volume of the convex hull occupied by species in trait space, representing the total functional space filled by a community. When plotted, we found that the convex hulls generated by antipatharians and scleractinians, respectively, were similar in size and shape and largely overlapped to generate the global functional trait space (Figure 5c).

### 3.5 Overall Significance of the Different Variables Driving the Fish Community Structure

The dbRDA analysis (PERMANOVA; pseudo‐*F* = 1.86, 999 permutations, *p* (perm) = 0.001) showed that coral taxon was the only variable that had a significant influence (*p* < 0.05) on the fish community (Appendix S2). For visualisation, the different variables added in the model (coral order, shelter volume and sites) were plotted as vectors according to the magnitude and direction of the relationship and overlaid on the fish community observed. Significant differences in the fish community between the coral taxa were found and confirmed by the one‐way permutation test (PERMANOVA; pseudo‐*F* = 3.13, 999 permutations, *p* (perm) = 0.001). The PERMDISP test confirmed equal dispersion within the two coral taxa (*F* = 0.97, *p* = 0.302).

## 4 Discussion

The ongoing decline of reefs globally has prompted greater interest in the functional roles of different reef‐associated taxa for preserving functional coral reef ecosystems (Bellwood et al. 2004; Darling et al. 2012; McLean et al. 2021). Nonetheless, studies of key ecological functions in corals (e.g., reef accretion and habitat provision) have focused mostly on scleractinians. Although reef accretion is mainly attributable to scleractinians and calcifying algae, other benthic taxa provide important habitat complexity that supports coral reef biodiversity and ecosystem functioning. Our study represents one of the first to examine the role of antipatharians in supporting fish communities on shallow tropical reef ecosystems. We provide empirical evidence for the contribution of antipatharians for habitat provision in shallow tropical reefs. We found that antipatharians support a diverse range of fish species that utilise them for a range of different purposes. They support unique fish species and functional entities and unique types of associations with fishes compared to scleractinians, but there are also overlaps between the coral taxa. At the functional level, fish assemblages display considerable similarity between antipatharians and scleractinians, suggesting that antipatharians could provide some redundancy of fish functional roles if scleractinians decline. Consequently, our findings underscore that, owing to their unique fish assemblages and the overlap with those linked to scleractinians, antipatharians serve as essential components of shallow tropical marine ecosystems.

### 4.1 Fish Communities Associated With Antipatharians

**FIGURE 3:**
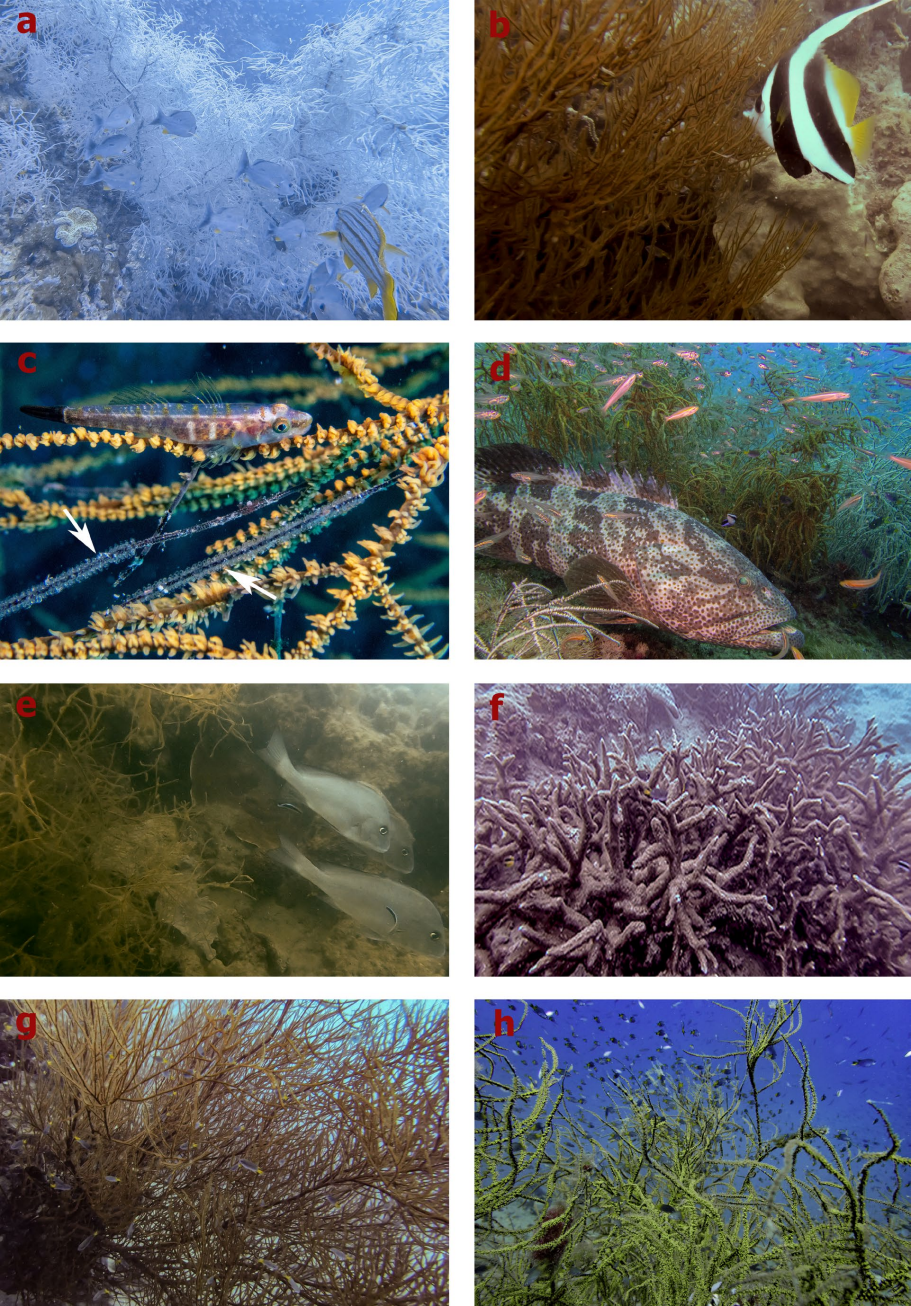
Examples of interactions between fish and antipatharians and scleractinians documented in this study: (a) *Lutjanus russellii* and *L. carponotatus* behind a white antipatharian colony sheltering from the current at *Yongala*. (b) *Heniochus acuminatus* feeding on the polyps of an antipatharian colony at Orpheus. (c) *Bryaninops tigris* residing on an antipatharian colony at *Yongala*; white arrows show its eggs deposited on the colony branches. (d) *Epinephelus fuscoguttatus* laying among antipatharian colonies at *Yongala*. (e) *Diagramma pictum* being cleaned by *Labroides dimidiatus* while hovering next to an antipatharian colony at Orpheus. (f) A range of fish species hiding among a branching scleractinian colony at Orpheus. (g) A range of fish species hiding among a branching antipatharian colony at Orpheus. (h) A range of fish species sheltering among a branching antipatharian at *Yongala* (Photos: Erika Gress).

There were 23 functional entities of the 28 different fish species associated with antipatharians, suggesting a high diversity of functional roles within their fish community. The most common and abundant species were primarily using the colonies as shelter (HovH, HovA behaviours; Figure 1c). This is not surprising considering the ample shelter capacity that branching corals provide for small‐bodied fish and/or juvenile fish (Beukers et al. 1997; Kerry and Bellwood 2015b), which is the most common morphology of antipatharians at both surveyed sites (Figure 3). This type of association (hovering around the colonies for protection) was not restricted to small‐bodied fish—at both sites, we also recorded larger fish (e.g., *Lutjanus russellii* , *L. carponotatus* , *Platax pinnatus* ) hovering behind antipatharian colonies (Figure 3a). These larger fish may be using the antipatharian colonies to shelter from strong currents or to ambush prey. Additionally, we documented corallivorous fish (e.g., *Chaetodon rainfordi* , *Heniochus acuminatus* ) feeding on antipatharian polyps (Figure 2b). We also observed several *Gobiodon* species using antipatharians as habitat (Figure 3c; see also Allen et al. 2004), but we were not able to quantify the abundance of these cryptic fish using MaxN through video analysis. Nonetheless, further studies of cryptic reef fishes and their symbiosis with antipatharians deserve attention due to their important role in coral reef energy transfer (Brandl, Tornabene, et al. 2019).

The use of antipatharians as nocturnal shelter by predator fishes has been reported from mesophotic reefs in Hawaii (Boland and Parrish 2005). In this current study conducted during daylight hours, we documented predator fishes (e.g., *Plectropomus leopardus* , *Cephalopholis boenak* ) laying static next to or under antipatharian colonies (Stat_in behaviour), which were potentially sheltering or waiting to ambush smaller fish. While not recorded during our stationary videos, we also observed other species of conservation interest and commercial value, such as the marble‐grouper ( *Epinephelus fuscoguttatus* ), laying static among antipatharian colonies (Figure 3d). In addition, cleaner wrasse ( *Labroides dimidiatus* )—which uses the antipatharians as habitat and refuge—attracted larger fish (e.g., *Diagramma pictum labiosum* , *Platax teira* ) which hovered next to the coral colonies to get cleaned (Figure 3e).

Predator–prey interactions are considered one of the most fundamental ecological dynamics on coral reefs (Hixon and Beets 1993). In the present study, we observed several large predator fish (e.g., *Carangoides fulvoguttaus*, *Plectropomus maculatus* , *Lutjanus monostigma* , and so forth) passing by; and despite these fishes being likely foraging, we did not observe any actual predation events on the stationary cameras due to their limited field of view. Nonetheless, schools of the greater amberjack ( *Seriola dumerili* ), the bluefin tuna (*Tunnus thynnus*) and the yellowmouth barracuda ( *Sphyraena viridensis* ) have been documented searching for fish prey among antipatharian forests on TMEs in the Mediterranean (Chimienti et al. 2020). Consequently, antipatharians are important for a range of fish species, providing both protection for prey and foraging opportunities for predators.

### 4.2 Influence of the Different Variables on the Fish Community Observed

#### 4.2.1 Site

**FIGURE 4:**
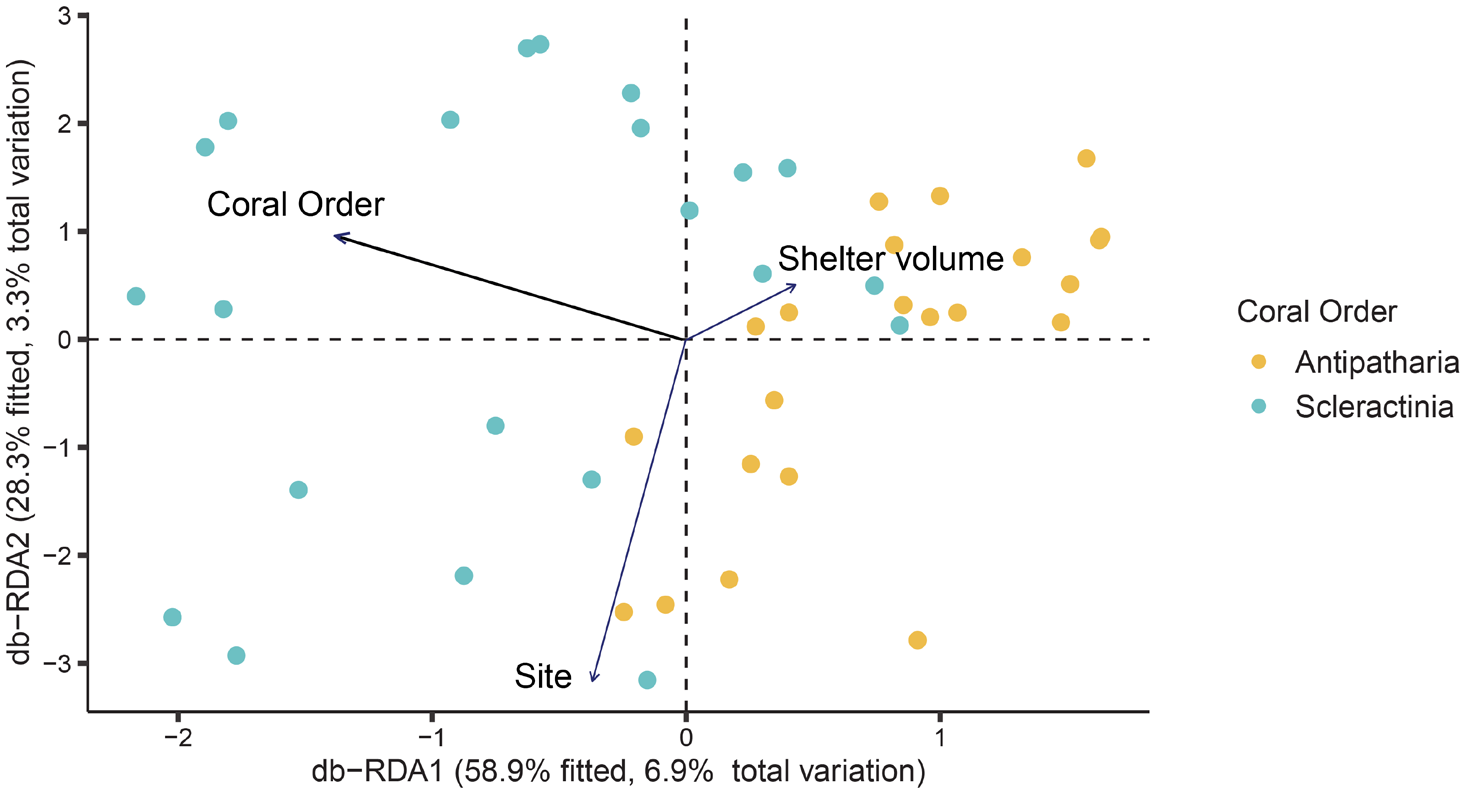
Distance‐based redundancy analysis (dbRDA) of fish communities associated to antipatharian (yellow dots) and scleractinian (blue dots) colonies. Vectors (arrows) represent the different variables tested on their significance as drivers of the fish community. The length and direction of the arrow represents the magnitude and direction of the relationship. Coral taxon (thicker arrow) was identified as the only significant variable (*p* < 0.05) influencing the fish community (Appendix S2).

We found site to have a significant effect on the fish density m^−2^ and richness m^−2^ (Table 1), which was not unexpected considering that fish communities on shipwrecks are known to differ from those found on natural reefs (Nieves‐Ortiz et al. 2021; Sánchez‐Caballero et al. 2021). Nonetheless, differences in fish richness and density are also evident from studies comparing coral reefs with both similar and distinct topographies, and the differences are often driven by site‐level factors (Galbraith et al. 2021, 2023; Gilby et al. 2016). Despite fish density m^−2^ and richness m^−2^ being higher at *Yongala* (Figures 1 and 2), our dbRDA analysis of fish community composition—testing the influence of sites, coral taxa and shelter volume—revealed no significant effect of sites on community structure (Figure 4). This suggests that site‐specific factors did not significantly shape overall fish community composition.

At *Yongala*, depth did not have a significant effect on the fish community, and most of the fish recorded occur across the wreck depth gradient (14–29 m depth). The one exception was *Ostorhinchus cladophilos*, which is not typically found above 20 m depth (Froese and Pauly 2024). Sixty‐two per cent of the fish recorded in this study inhabit mesophotic ecosystems, slightly lower than a study from Hawaii that reported that 95% of the fish documented in association with antipatharians on mesophotic reefs also occur on shallow reefs (Boland and Parrish 2005). Nonetheless, in the eastern Atlantic—where antipatharian forests are found at mesophotic depths—the most common functional entities and species shifted between shallow and mesophotic reefs, even when 90% of the fish functional entities were shared between shallow and mesophotic reefs (Bosch et al. 2023). Thus, antipatharians might promote specialisation of reef fishes along the reef depth gradient (Bosch et al. 2023), which is yet to be investigated on tropical reefs.

#### 4.2.2 Area and Shelter Volume

In addition to colony area (m^2^), we used shelter volume (dm^−3^) to quantify one of the most important ecological functions of corals—shelter provision (Urbina‐Barreto et al. 2021, 2022). Importantly, shelter volume encompasses both the area of the coral and its morphology, both of which influence specific ecological functions (Kerry and Bellwood 2015a; Lingo and Szedlmayer 2006). In light of the lack of proxies to estimate shelter volume specifically for antipatharians, we use the ones developed for scleractinians (Urbina‐Barreto et al. 2021) considering that ‘branching’ morphology of scleractinians and antipatharians varies in terms of branch thickness and arrangement, and antipatharians tend to exhibit more intricate and complex structures. As such, applying the same morphological proxies to both coral groups is a conservative approach. Indeed, our results suggest that these proxies adequately capture shelter volume in antipatharians. Notably, our models results were similar regardless of whether area or shelter volume was used. Nonetheless, dedicated proxies for antipatharians would be preferable for future studies.

Structural complexity is a key predictor of both fish abundance and species richness on coral reefs (Darling et al. 2017; Graham and Nash 2013; Urbina‐Barreto et al. 2022). However, in our study, shelter volume influenced fish richness, but not abundance. Despite greater shelter volume comprising more habitat, niche space within a colony is more homogenous than at the colony perimeter (Boström‐Einarsson et al. 2014; Holbrook and Schmitt 2002; Robertson 1996). Therefore, our results could be related to large colonies—with homogenous internal shelter volume—regulating fish abundance through competitive interactions. Additionally, nuanced relationships between patch habitat area and edge interactions with surrounding habitats are often associated with species richness, but not to abundance (Fonseca 2008; Hattori and Shibuno 2015). For instance, fish species richness may be enhanced around the colony perimeter where the habitat is more complex and where opportunities for interactions with surrounding habitat are optimised (Hattori and Shibuno 2015).

#### 4.2.3 Coral Taxon

**FIGURE 5:**
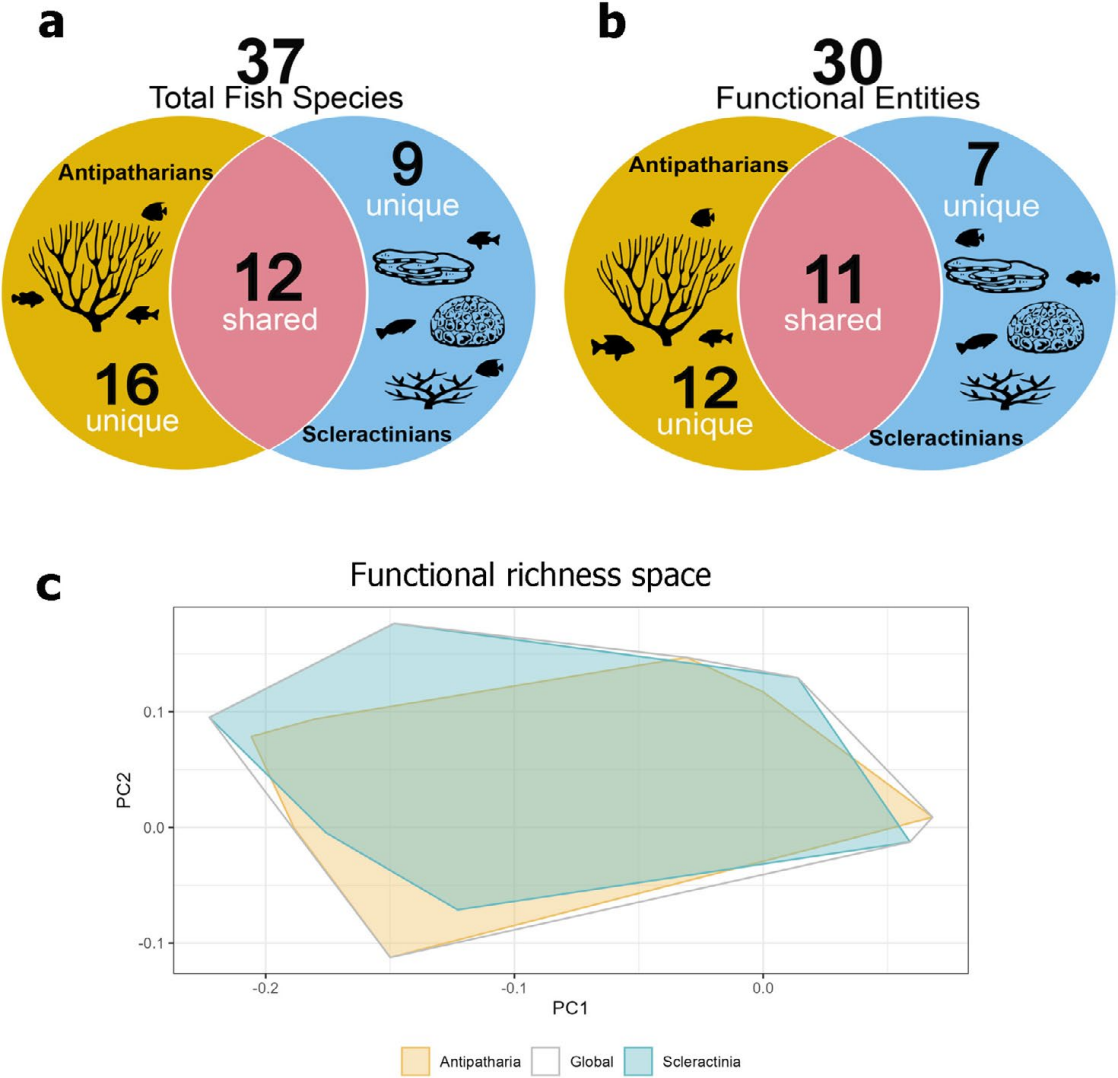
Fish communities associated with antipatharians and scleractinians. (a) Unique and shared fish species associated to each coral taxon. (b) Unique and shared functional entities associated to each coral taxon. (c) Coral‐associated fish functional richness space illustrating the distribution of functional richness for antipatharians (yellow hull), scleractinians (blue hull) and the global (i.e., overall, white hull).

The density m^−2^, and number of functional entities of fish communities varied significantly between antipatharians and scleractinians; nonetheless, there was not a significant difference in species richness m^−2^ and both coral taxa supported seven functional groups (Figures 1c and 5). There is high overlap of fish species associated with both coral taxa, and despite some species found in unique association with either scleractinians or antipatharians (Figure 5a), none of these species are considered as either antipatharian or scleractinian specialists (Froese and Pauly 2024). One potential explanation for the difference in fish richness among coral taxa is the type of association with the corals. For instance, while the most abundant families (Apogonidae, Pomacentridae, Labridae) were shared between both coral taxa, Lutjanidae was 80% more abundant for antipatharians (Figure 2a). Within the family Lutjanidae, *L. russellii* and *L. carponotatus* were only recorded in association with antipatharians, and both fish species appeared to be using the colonies as shelter from currents (HovH behaviour; Figure 3a). Similar specific interactions have been observed for scleractinians; for example, some fish use tabular *Acropora* colonies to protect themselves from solar irradiance (Kerry and Bellwood 2015b). Therefore, species‐specific associations may contribute to dissimilarities in the fish species associating with antipatharians and scleractinians.

At the functional level, fish communities associated with antipatharians exhibited significantly higher functional diversity, with a greater number of unique functional entities compared to those associated with scleractinians (Figure 5b), suggesting that antipatharians can support fish faunas with more varied ecological roles. In contrast, analysis of functional redundancy shows that antipatharian‐associated communities have slightly higher redundancy than the ones in association with scleractinians, meaning more species share similar ecological roles; although the difference was not significant. The functional space analysis illustrates these dynamics (Figure 5c), with antipatharian and scleractinian communities showing large overlap in their functional spaces, indicating shared ecological roles while also revealing distinct differences that underscore the broader functional range supported by each coral taxon. The hulls in the plot further emphasise this overlap and variation, highlighting the unique contributions of each coral taxa to the global functional space (Figure 5c). Although higher functional diversity implies more unique roles and higher redundancy suggests more overlapping roles, these findings are not necessarily contradictory but reflect complex ecological dynamics. Antipatharians may provide diverse niches that foster unique functional roles while also supporting multiple species within those roles, potentially enhancing resilience against species loss. Scleractinian‐associated communities, with fewer unique functional entities and slightly lower redundancy, may be more vulnerable to losing critical ecological functions. These findings, in the context of functional diversity and vulnerability in tropical reef fish faunas (Mouillot et al. 2014), highlight the critical influence of coral type on both the variety and stability of ecological roles in reef ecosystems.

Variation in fish communities may be in part attributable to intrinsic differences in morphological complexity between the two coral taxa. The differences in complexity and branching arrangement between antipatharians and scleractinians are shown in Figure 3f,g,h. Antipatharians do not grow as massive or encrusting colonies, and all growth forms extend vertically off the substrate, thereby increasing the exposed area available for habitat. Moreover, the canopy‐like effect created by most antipatharian growth forms can enhance fine‐scale hydrodynamic conditions (e.g., upwelling) that promote the retention of plankton and juvenile fish, which benefits planktivorous, invertivorous and piscivorous fish species (Guizien and Ghisalberti 2017). Additionally, habitat spaces provided by densely branched colonies might also influence fish density due to the schooling behaviour of most planktivore fishes, and the refuge availability and survivorship for juvenile and small‐bodied fish. Although shelter volume provides a quantitative measure of the space available for shelter, it is based on colony area or diameter (Urbina‐Barreto et al. 2021); therefore, it does not capture the elevation from the substrate (colony height). This could explain why neither shelter volume nor area had a significant effect on fish abundance.

Numerous studies have identified colony height as a more influential factor driving fish assemblages than surface area or coral shape (Fisher 2023; Harborne et al. 2012). Therefore, future studies should quantify both shelter volume and colony height of corals when examining their correlation with fish assemblages. Additionally, the development of proxies specifically for antipatharians could enable finer‐scale morphological differences to be captured. This information will enable trait‐based approaches to understanding coral reef function to be extended to a wider range of benthos, rather than just scleractinians—an important approach considering scleractinians are not necessarily the dominant habitat‐forming benthos in many shallow tropical ecosystems.

### 4.3 Implications for Conservation

The importance of trait‐based approaches to support and guide local and regional conservation strategies in light of the current coral reefs crisis is now well recognised (Bellwood et al. 2004; Hughes et al. 2017; McLean et al. 2021). However, most studies utilising trait‐based approaches in coral reef ecology and the influence of benthic communities on fish assemblages focus on scleractinians (Darling et al. 2017; Fisher 2023; Harborne et al. 2012). Our study highlights that other coral taxa can significantly influence reef fish communities, playing an important role in providing three‐dimensional habitat complexity on shallow tropical reefs. Other habitat‐forming benthic groups have been previously considered (e.g., octocorals and sponges; González‐Murcia et al. 2023; Moynihan et al. 2022); however, antipatharians are commonly neglected from coral reef monitoring programs and studies. A greater effort to quantify the abundance and ecological roles of the different benthic groups would lead to a more holistic understanding of how the different benthic taxa interact to support coral reef biodiversity.

While antipatharians are not abundant in the shallowest depths (< 10 m), they are common in most other reef depths, in both shallow and mesophotic reefs (Molodtsova et al. 2023; Wagner et al. 2012). Importantly, antipatharians are less susceptible to the phenomenon known as bleaching (Gress et al. 2021) and other climate‐related stressors (Godefroid et al. 2023) than scleractinians. Given the impact of bleaching events on scleractinians (Hughes et al. 2017, 2018), the importance of other coral taxa in supporting and maintaining reef ecological functions requires a greater understanding to account for in conservation strategies.

Human activities such as fisheries have led to some antipatharian species being listed as ‘near threatened’ by the International Union for Conservation of Nature (IUCN) Red List of the Mediterranean (Bo et al. 2008, 2017). Nonetheless, the status of antipatharian species outside the Mediterranean remains unknown despite evidence of declines on some tropical reefs (Boland and Parrish 2005; Gress and Kaimuddin 2021; Grigg 2004). Considering the relevance of antipatharians in supporting reef biodiversity, we argue that a greater effort should be afforded to understanding the role of antipatharians and their status worldwide.

## Author Contributions

**Erika Gress:** conceptualization (lead), data curation (lead), formal analysis (lead), funding acquisition (lead), investigation (lead), methodology (lead), project administration (lead), writing – original draft (lead), writing – review and editing (equal). **Kevin R. Bairos‐Novak:** formal analysis (equal), writing – review and editing (equal). **Tom C. Bridge:** funding acquisition (equal), writing – review and editing (equal). **Gemma F. Galbraith:** data curation (equal), formal analysis (equal), methodology (equal), writing – review and editing (equal).

## Conflicts of Interest

The authors declare no conflicts of interest.

## Supporting information


**Appendix S1:** ece372015‐sup‐0001‐AppendixS1.pdf.

**Appendix S2:** ece372015‐sup‐0002‐AppendixS2.pdf.

**Appendix S3:** ece372015‐sup‐0003‐AppendixS3.pdf.

## Data Availability

Appendix 1 contains the datasets generated during the current study available via the following link: https://figshare.com/s/f0f6d8fc855a81628b66. Appendix 2 contains the dataset generated for fish functional analyses available via the following link: https://figshare.com/s/da1b6baa16db0c0c47f8.
