# Supplementary material for: Neglected Biodiversity of Fish Assemblages Associated With Antipatharia (Black Corals) on Tropical Shallow Reef Ecosystems
Source: Ecol Evol. 2025 Aug 21;15(8):e72015. doi: 10.1002/ece3.72015 (PMC12370845; doi:10.1002/ece3.72015)
Supplement: Supplementary file 1 — Appendix S1: ece372015‐sup‐0001‐AppendixS1.pdf. [file ECE3-15-e72015-s001.pdf]

Supplementary 1a. Map of the study sites: *SS Yongala* and Orpheus Island. Sites are ~142 km apart and are both on the central Great Barrier Reef, in Queensland Australia.

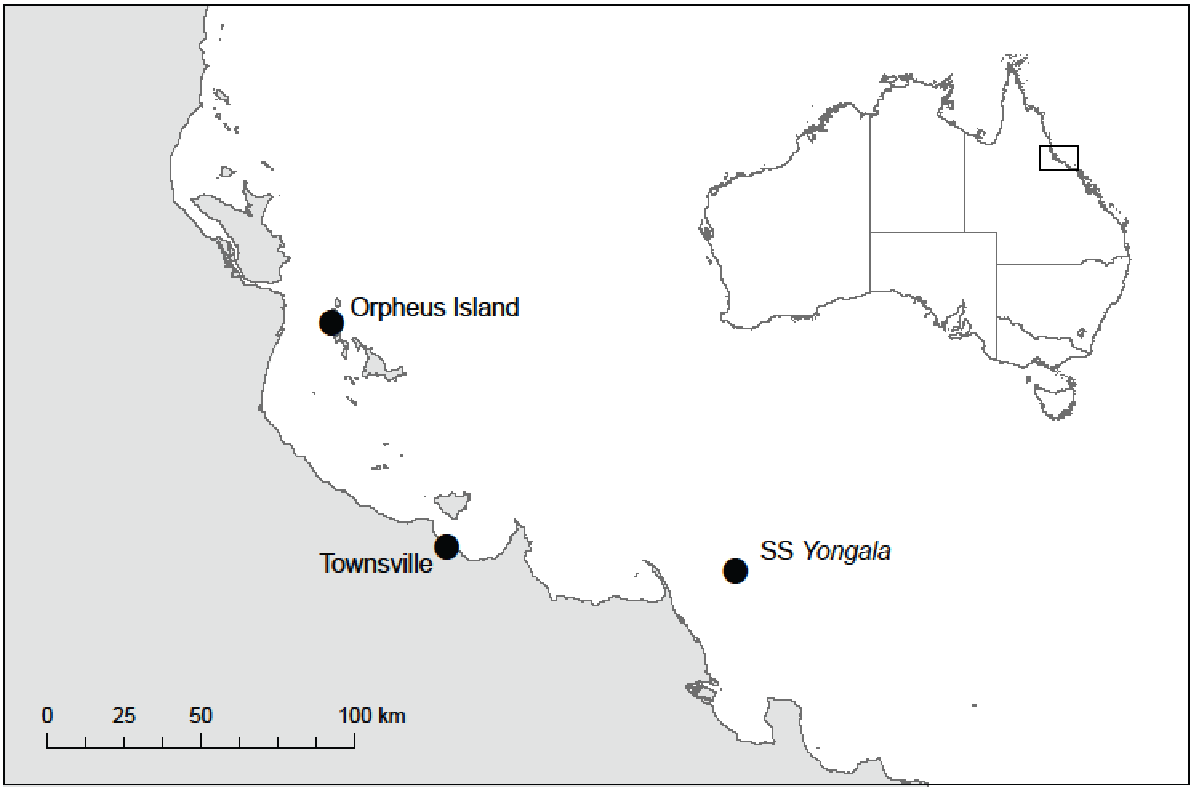

Supplementary 1b. Linear models of the shelter volume (dm^3) of the antipatharian and scleractinian corals as a function of diameter (top) and planar area (bottom) - based on proxies developed by Urbina-Barreto et al. (2021). For antipatharians, the area was calculated based on width (diameter) and height and of the colonies as view from the side, which are the best estimators of surface area for non-scleractinian branching bushy-like coral colonies (Santavy et al. 2013). Growth forms: Branching (orange); Tabular (olive green); and Massive (green). Encrusting (blue) and Foliose (pink) proxies are not avaible; therefore they were treated as Massive following Urbina-Barreto et al. (2021). Confidence intervals (95%) are represented by light colored bands.

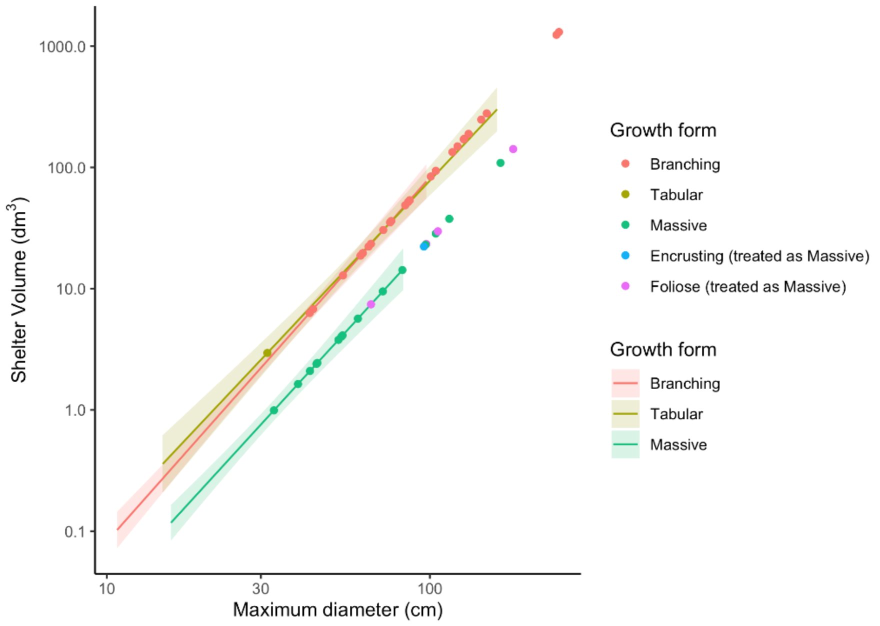

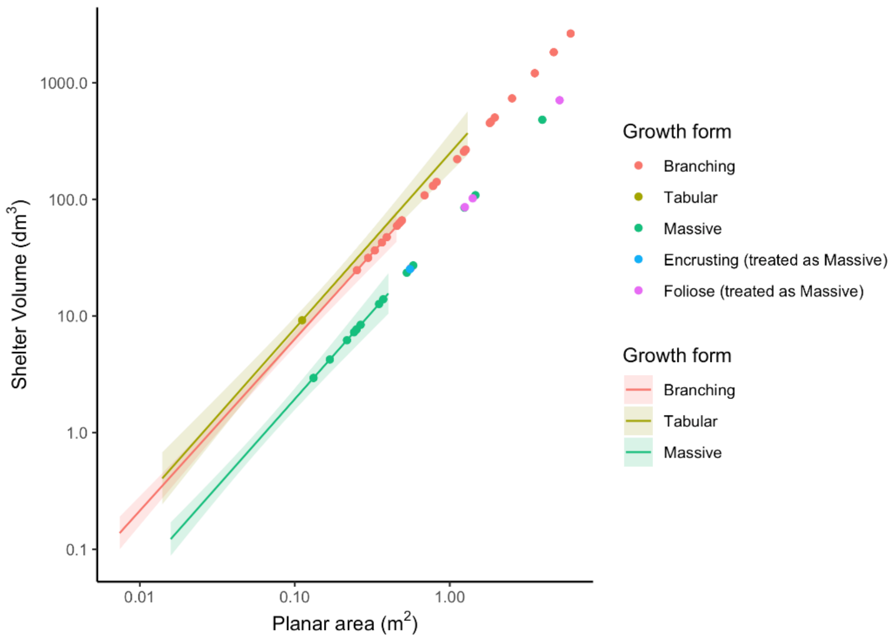

- **Antipatharia growth form**

Branching

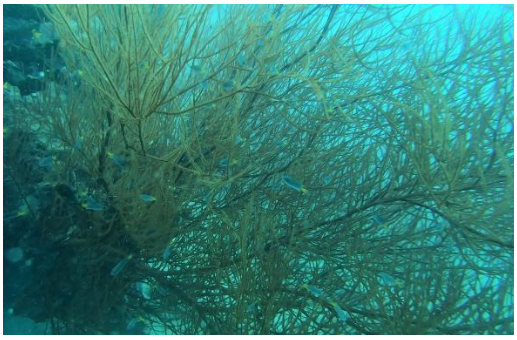

- **Scleractinia growth forms**

Branching Tabular Massive Foliose

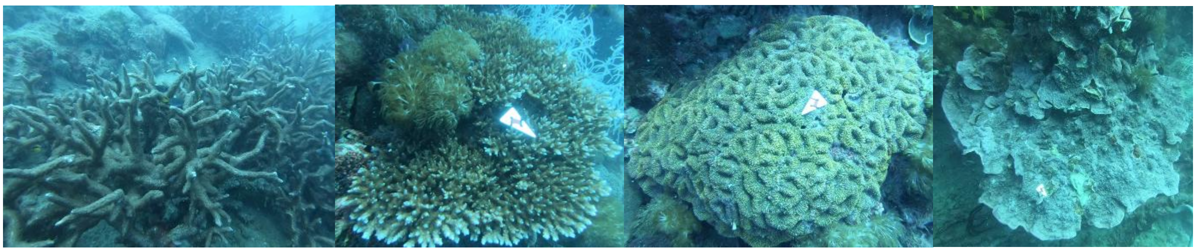

Supplementary 1c. Relative fish species richness m^{-2} for each fish family.

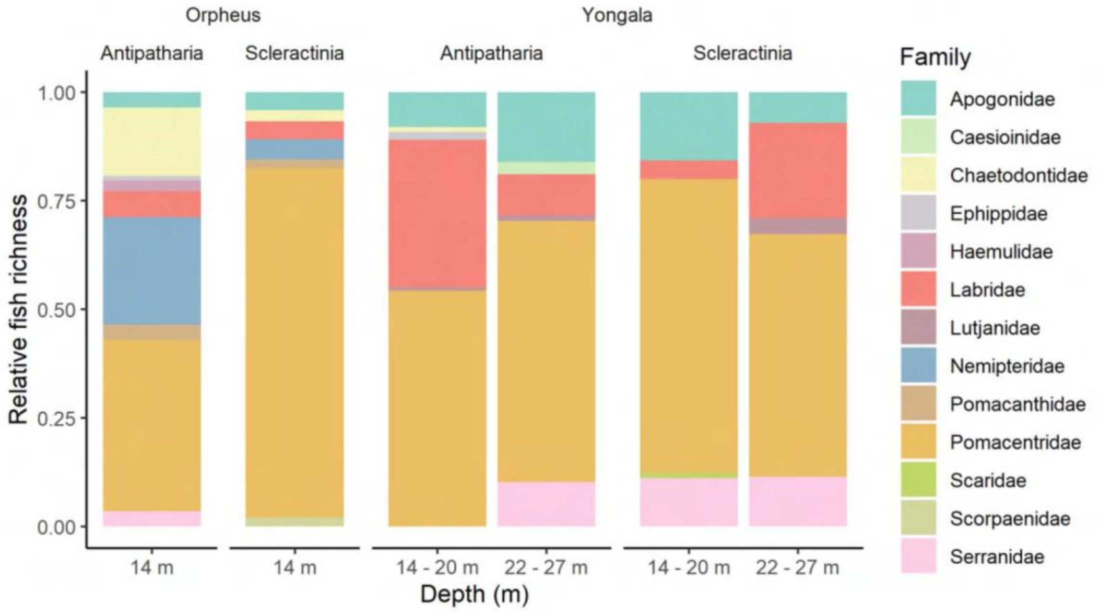

Orpheus
Yongala
Antipatharia
Scleractinia
Antipatharia
Scleractinia
Relative fish richness
1.00
0.75
0.50
0.25
0.00
14 m
14 m
14 - 20 m
22 - 27 m
14 - 20 m
22 - 27 m
Depth (m)
Family
Apogonidae
Caesioinidae
Chaetodontidae
Ephippidae
Haemulidae
Labridae
Lutjanidae
Nemipteridae
Pomacanthidae
Pomacentridae
Scaridae
Scorpaenidae
Serranidae
