# Supplementary material for: Neglected Biodiversity of Fish Assemblages Associated With Antipatharia (Black Corals) on Tropical Shallow Reef Ecosystems
Source: Ecol Evol. 2025 Aug 21;15(8):e72015. doi: 10.1002/ece3.72015 (PMC12370845; doi:10.1002/ece3.72015)
Supplement: Supplementary file 2 — Appendix S2: ece372015‐sup‐0002‐AppendixS2.pdf. [file ECE3-15-e72015-s003.pdf]

Supplementary 2

Gress et al. 2025

Data import and preparation

```
final_data <- read_csv("../2_outputs/final_data.csv") %>%
  mutate(
    PAIR = as.factor(PAIR),
    SITE = as.factor(SITE),
    DEPTH_RANGE = as.factor(DEPTH_RANGE),
    CORAL_ORDER = as.factor(CORAL_ORDER),
    CORAL_MORPHO = as.factor(CORAL_MORPHO),
    COLONY_N = as.factor(COLONY_N),
    U_ID = as.factor(U_ID)
  )

# Remove fish data (coral colony-specific data):
data_distinct <- final_data %>%
  dplyr::select(-c(FISH_GENUS:BINOMIAL, Genus:Vulnerability, `Trophic
Group`:`Group Size`, Regions)) %>%
  distinct()
```

There are two distinct depths at the Yongala site and one distinct and shallower depth at Orpheus; therefore, any model looking at the effect of depth precludes including the Orpheus site in the analysis.

```
data_distinct %>%
  ggplot(aes(x = DENSITY_m2, color = CORAL_ORDER, fill = CORAL_ORDER)) +
  facet_grid(SITE ~ DEPTH_RANGE) +
  geom_histogram()
```

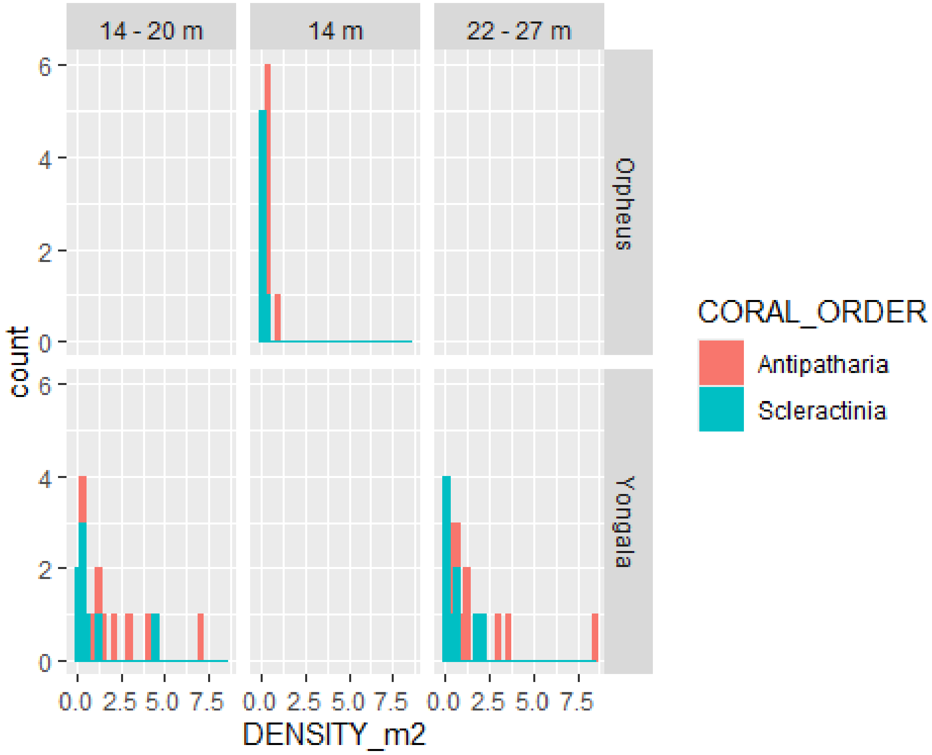

Therefore, we subset by Yongala, and further subset by antipatharian vs. scleractinian corals.

```
# Subset data by site
data_yong <- data_distinct %>% filter(SITE == "Yongala")
data_yong_anti <- data_yong %>% filter(CORAL_ORDER == "Antipatharia")
data_yong_sclera <- data_yong %>% filter(CORAL_ORDER == "Scleractinia")
```

MODEL 1 - Does **depth** affect **fish density per area** between the two depth bands at Yongala?

```
glmm1 <- glmmTMB(DENSITY_m2 ~ DEPTH_RANGE * CORAL_ORDER + (1 | PAIR),
  family = Gamma(link = "log"), data = data_yong,
  REML = TRUE, na.action = na.omit
)
nrow(data_yong)

## [1] 34

glmm1 %>% simulateResiduals(plot = TRUE)
```

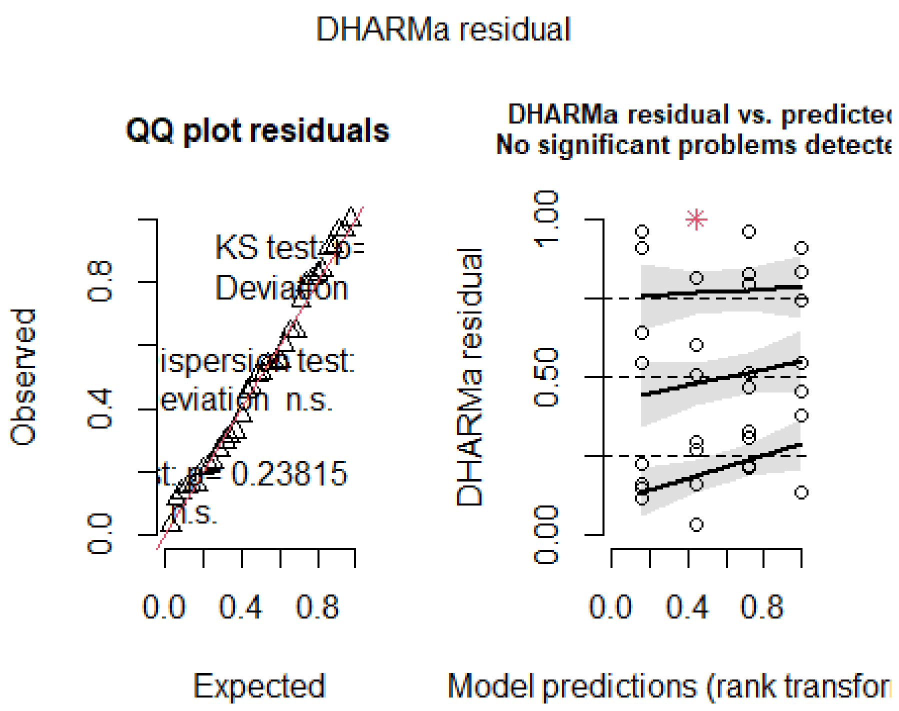


```
## Object of Class DHARMa with simulated residuals based on 250 simulations
with refit = FALSE . See ?DHARMa::simulateResiduals for help.
##
## Scaled residual values: 0.38 0.508 0.136 0.604 0.456 0.272 0.544 0.032
0.22 0.964 0.328 0.644 0.212 0.644 0.312 0.908 0.796 0.116 0.912 0.296 ...

Anova(glmm1)

## Analysis of Deviance Table (Type II Wald chisquare tests)
##
## Response: DENSITY_m2
##                          Chisq Df Pr(>Chisq)
## DEPTH_RANGE             0.0639  1   0.800380
## CORAL_ORDER             9.5761  1   0.001971 **
## DEPTH_RANGE:CORAL_ORDER 0.0000  1   0.999713
## ---
## Signif. codes:  0 '***' 0.001 '**' 0.01 '*' 0.05 '.' 0.1 ' ' 1

# Parameter significance:
summary(glmm1)

##  Family: Gamma  ( log )
## Formula:          DENSITY_m2 ~ DEPTH_RANGE * CORAL_ORDER + (1 | PAIR)
## Data: data_yong
##
##      AIC      BIC   logLik deviance df.resid
##     97.6    106.7    -42.8     85.6       28
```

```
##
## Random effects:
##
## Conditional model:
##  Groups Name        Variance Std.Dev.
##  PAIR   (Intercept) 0.1961   0.4428
## Number of obs: 34, groups:  PAIR, 17
##
## Dispersion estimate for Gamma family (sigma^2): 1.09
##
## Conditional model:
##                                                Estimate Std. Error z value
## (Intercept)                                   0.7987360  0.4189321   1.907
## DEPTH_RANGE22 - 27 m                         -0.1107615  0.5711974  -0.194
## CORAL_ORDERScleractinia                      -1.2289974  0.5724098  -2.147
## DEPTH_RANGE22 - 27 m:CORAL_ORDERScleractinia  0.0002813  0.7810093   0.000
##                                              Pr(>|z|)
## (Intercept)                                    0.0566 .
## DEPTH_RANGE22 - 27 m                           0.8462
## CORAL_ORDERScleractinia                        0.0318 *
## DEPTH_RANGE22 - 27 m:CORAL_ORDERScleractinia   0.9997
## ---
## Signif. codes:  0 '***' 0.001 '**' 0.01 '*' 0.05 '.' 0.1 ' ' 1
```

```
emmeans::emmeans(glmm1, ~ DEPTH_RANGE + CORAL_ORDER, type = "response")
```

```
##  DEPTH_RANGE CORAL_ORDER  response    SE  df asymp.LCL asymp.UCL
##  14 - 20 m   Antipatharia    2.223 0.931 Inf     0.978      5.05
##  22 - 27 m   Antipatharia    1.990 0.796 Inf     0.908      4.36
##  14 - 20 m   Scleractinia    0.650 0.317 Inf     0.250      1.69
##  22 - 27 m   Scleractinia    0.582 0.240 Inf     0.260      1.30
##
## Confidence level used: 0.95
## Intervals are back-transformed from the log scale
```

No significant effect of depth detected, only coral order!

MODEL 2 - Does **depth** affect **fish richness per area** between the two depth bands at Yongala?

```
glmm2 <- glmmTMB(RICHNESS_m2 ~ DEPTH_RANGE * CORAL_ORDER + (1 | PAIR),
  family = Gamma(link = "log"), data = data_yong,
  REML = TRUE, na.action = na.omit
)
nrow(data_yong)
```

```
## [1] 34
```

```
glmm2 %>% simulateResiduals(plot = TRUE) # model looks good
```

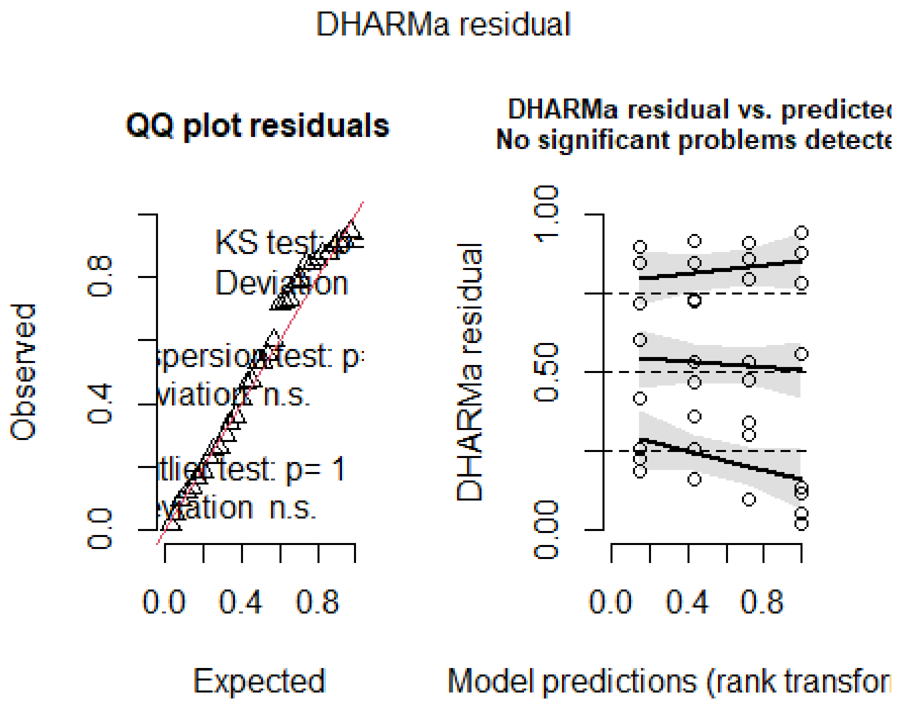


```
## Object of Class DHARMa with simulated residuals based on 250 simulations
with refit = FALSE . See ?DHARMa::simulateResiduals for help.
##
## Scaled residual values: 0.856 0.6 0.472 0.716 0.912 0.256 0.532 0.188
0.468 0.944 0.916 0.876 0.26 0.784 0.728 0.876 0.844 0.12 0.796 0.228 ...
```

```
Anova(glmm2)
```

```
## Analysis of Deviance Table (Type II Wald chisquare tests)
##
## Response: RICHNESS_m2
##                           Chisq Df Pr(>Chisq)
## DEPTH_RANGE              0.4934  1   0.482424
## CORAL_ORDER              0.5301  1   0.466574
## DEPTH_RANGE:CORAL_ORDER 6.7256  1   0.009504 **
## ---
## Signif. codes:  0 '***' 0.001 '**' 0.01 '*' 0.05 '.' 0.1 ' ' 1
```

```
summary(glmm2)
```

```
##  Family: Gamma  ( log )
## Formula:          RICHNESS_m2 ~ DEPTH_RANGE * CORAL_ORDER + (1 | PAIR)
## Data: data_yong
##
##      AIC      BIC   logLik deviance df.resid
##    -99.4    -90.2     55.7   -111.4       28
##
```

```
## Random effects:
##
## Conditional model:
##  Groups Name        Variance  Std.Dev.
##  PAIR   (Intercept) 1.558e-09 3.948e-05
## Number of obs: 34, groups:  PAIR, 17
##
## Dispersion estimate for Gamma family (sigma^2): 0.581
##
## Conditional model:
##                                              Estimate Std. Error z value
## (Intercept)                                   -2.4206     0.2694  -8.985
## DEPTH_RANGE22 - 27 m                          -0.4951     0.3702  -1.337
## CORAL_ORDERScleractinia                       -0.5286     0.3810  -1.388
## DEPTH_RANGE22 - 27 m:CORAL_ORDERScleractinia   1.3579     0.5236   2.593
##                                              Pr(>|z|)
## (Intercept)                                    <2e-16 ***
## DEPTH_RANGE22 - 27 m                           0.1812
## CORAL_ORDERScleractinia                        0.1653
## DEPTH_RANGE22 - 27 m:CORAL_ORDERScleractinia   0.0095 **
## ---
## Signif. codes:  0 '***' 0.001 '**' 0.01 '*' 0.05 '.' 0.1 ' ' 1
```

```
emmeans::emmeans(glmm2, ~ DEPTH_RANGE + CORAL_ORDER, type = "response")
```

```
##  DEPTH_RANGE CORAL_ORDER  response     SE  df asymp.LCL asymp.UCL
##  14 - 20 m   Antipatharia   0.0889 0.0239 Inf    0.0524    0.1507
##  22 - 27 m   Antipatharia   0.0542 0.0138 Inf    0.0329    0.0891
##  14 - 20 m   Scleractinia   0.0524 0.0141 Inf    0.0309    0.0888
##  22 - 27 m   Scleractinia   0.1241 0.0315 Inf    0.0755    0.2042
##
## Confidence level used: 0.95
## Intervals are back-transformed from the log scale
```

Significant interaction effect detected, but there is no additive effect of depth range or coral taxon.

To determine the significance of this interaction, we:

1. examine confidence intervals across coral order vs. depth range
2. examine pairwise contrasts of coral order vs. depth range, and
3. split up and conduct separate subanalyses for antipatharia vs. scleractinia to get the depth effect per taxon

In each of these cases, the end result is a non-significant effect of depth range, suggesting that this interaction is marginally significant and not well supported by the data.

1. Plot the confidence interval estimates:

```
# Calculate confidence limits and plot with data:
emmeans::emmeans(glmm2, ~ DEPTH_RANGE + CORAL_ORDER, type = "response") %>%
  as.data.frame() %>%
  ggplot(aes(x = DEPTH_RANGE, y = response, color = CORAL_ORDER)) +
  geom_linerange(aes(ymin = asymp.LCL, ymax = asymp.UCL),
    position = position_dodge(width = 0.9)
  ) +
  geom_point(position = position_dodge(width = 0.9), size = 4) +
  geom_point(
    data = data_yong, aes(y = RICHNESS_m2),
    position = position_jitterdodge(
      jitter.width = 0.1, jitter.height = 0,
      dodge.width = 0.9, seed = 9
    ),
    alpha = 0.3
  ) +
  scale_color_manual(values = c("#F0C165", "#65BAC4")) +
  labs(
    x = "Depth range",
    y = expression("Richness (species m"^-2 * ")"),
    color = "Coral taxon"
  )
```

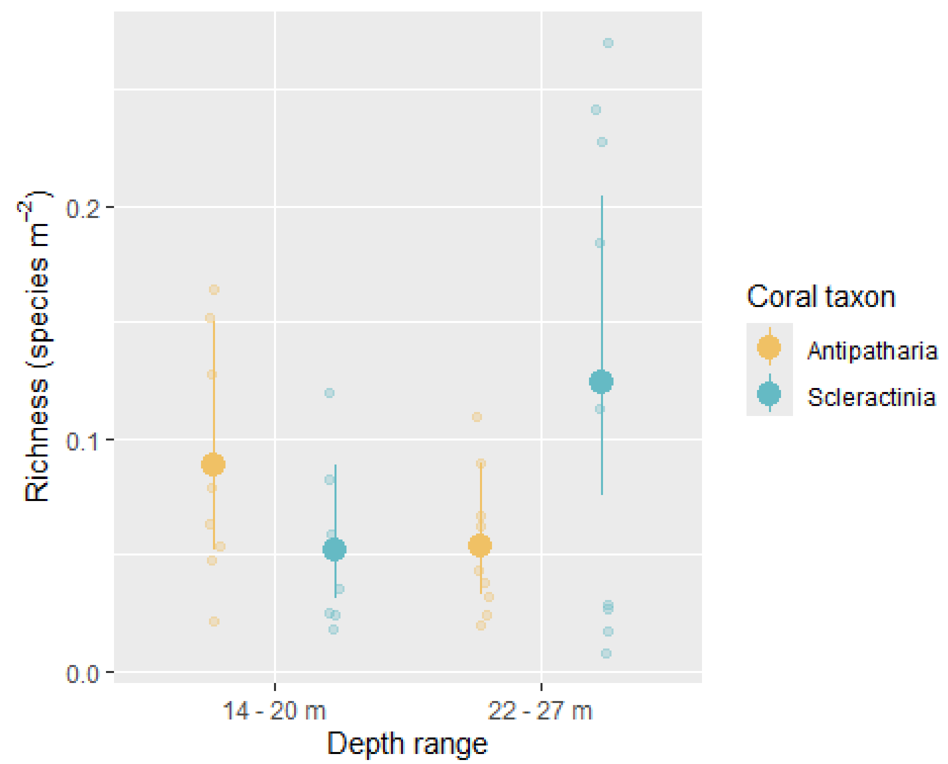

Note the overlap of all confidence limits, suggesting only marginal differences likely driven by some colonies of Scleractinia having very high richness and higher variability.

2. For the confidence intervals / significance of different pairwise factor combinations, we use the following:

```
# Pairwise contrasts:
glmm2 %>%
  emmeans::emmeans(pairwise ~ DEPTH_RANGE + CORAL_ORDER, type = "response")
%>%
  `[`("contrasts") %>%
  as.data.frame() %>%
  arrange(contrasts.p.value) %>%
  mutate(is_signif = ifelse(contrasts.p.value < 0.05, "*", ""))

##                                     contrasts.contrast contrasts.ratio
## 1 (14 / 20 m Scleractinia) / (22 / 27 m Scleractinia)       0.4219649
## 2 (22 / 27 m Antipatharia) / (22 / 27 m Scleractinia)       0.4363625
## 3 (14 / 20 m Antipatharia) / (14 / 20 m Scleractinia)       1.6965639
## 4 (14 / 20 m Antipatharia) / (22 / 27 m Antipatharia)       1.6405863
## 5 (14 / 20 m Antipatharia) / (22 / 27 m Scleractinia)       0.7158904
## 6 (22 / 27 m Antipatharia) / (14 / 20 m Scleractinia)       1.0341205
##   contrasts.SE contrasts.df contrasts.null contrasts.z.ratio
contrasts.p.value
## 1    0.1562278          Inf              1       -2.33047634
0.09118194
## 2    0.1567347          Inf              1       -2.30879048
0.09598867
## 3    0.6463446          Inf              1        1.38751389
0.50716985
## 4    0.6074090          Inf              1        1.33711945
0.53916999
## 5    0.2650505          Inf              1       -0.90273632
0.80338197
## 6    0.3828717          Inf              1        0.09062057
0.99973353
##   is_signif
## 1
## 2
## 3
## 4
## 5
## 6
```

Note that no pairwise contrasts are significant after applying Tukey HSD contrast correction, again suggesting a trending effect of an interaction, but no significant difference upon examining individual pairwise differences across Scleractinia vs. Antipatharia in terms of depth.

3. We now subset the analysis by coral taxon (Antipatharia and Scleractinia) separately to examine this interaction another way.

MODEL 2a - Does **depth** has an effect on **fish density m2** within **Antipatharia** between the two depth bands at Yongala?

```
glm2a_anti <- glmmTMB(RICHNESS_m2 ~ DEPTH_RANGE,
  family = Gamma(link = "log"), data = data_yong_anti,
  REML = TRUE, na.action = na.omit
)
nrow(data_yong_anti)

## [1] 17

glm2a_anti %>% simulateResiduals(plot = TRUE)

## Warning in smooth.construct.tp.smooth.spec(object, dk$data, dk$knots):
basis dimension, k, increased to minimum possible
## Warning in smooth.construct.tp.smooth.spec(object, dk$data, dk$knots):
basis dimension, k, increased to minimum possible
## Warning in smooth.construct.tp.smooth.spec(object, dk$data, dk$knots):
basis dimension, k, increased to minimum possible
```

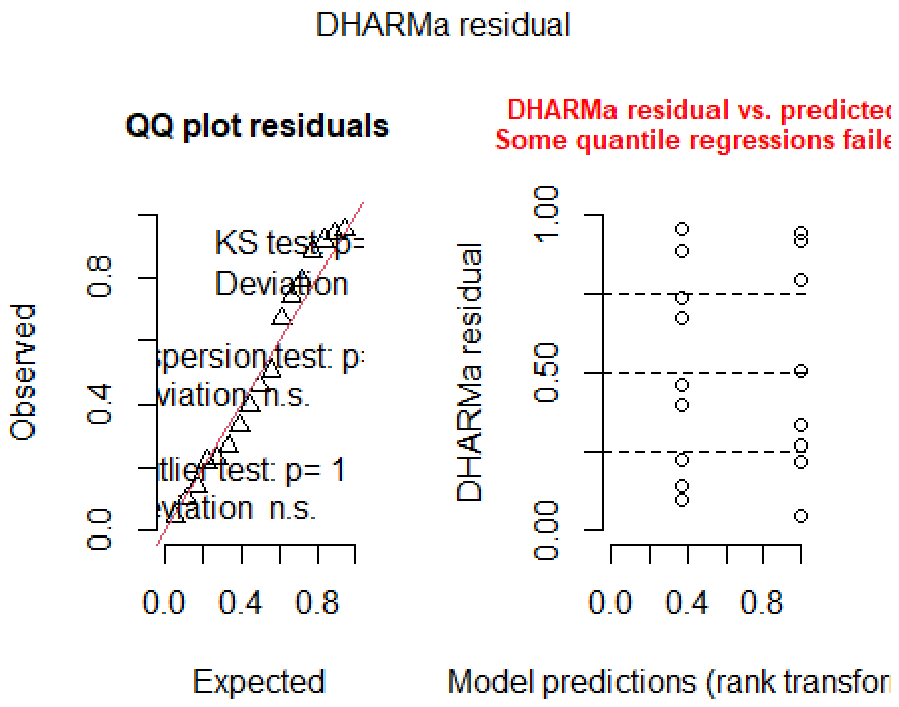


```
## Object of Class DHARMa with simulated residuals based on 250 simulations
with refit = FALSE . See ?DHARMa::simulateResiduals for help.
##
```

```
## Scaled residual values: 0.916 0.332 0.94 0.508 0.396 0.952 0.144 0.672
0.884 0.792 0.268 0.22 0.044 0.74 0.228 0.46 0.1

leveneTest(residuals(glm2a_anti) ~ data_yong_anti$DEPTH_RANGE) # non-
significant

## Levene's Test for Homogeneity of Variance (center = median)
##       Df F value Pr(>F)
## group  1  1.9421 0.1837
##       15

Anova(glm2a_anti)

## Analysis of Deviance Table (Type II Wald chisquare tests)
##
## Response: RICHNESS_m2
##             Chisq Df Pr(>Chisq)
## DEPTH_RANGE 3.001  1    0.08322 .
## ---
## Signif. codes:  0 '***' 0.001 '**' 0.01 '*' 0.05 '.' 0.1 ' ' 1

summary(glm2a_anti)

##  Family: Gamma  ( log )
## Formula:          RICHNESS_m2 ~ DEPTH_RANGE
## Data: data_yong_anti
##
##      AIC      BIC   logLik deviance df.resid
##    -57.9    -55.4     31.9    -63.9       14
##
##
## Dispersion estimate for Gamma family (sigma^2): 0.346
##
## Conditional model:
##                      Estimate Std. Error z value Pr(>|z|)
## (Intercept)           -2.4206     0.2079 -11.641   <2e-16 ***
## DEPTH_RANGE22 - 27 m  -0.4951     0.2858  -1.732   0.0832 .
## ---
## Signif. codes:  0 '***' 0.001 '**' 0.01 '*' 0.05 '.' 0.1 ' ' 1

emmeans::emmeans(glm2a_anti, ~DEPTH_RANGE, type = "response")

##  DEPTH_RANGE response     SE  df asymp.LCL asymp.UCL
##  14 - 20 m     0.0889 0.0185 Inf    0.0591    0.1336
##  22 - 27 m     0.0542 0.0106 Inf    0.0369    0.0795
##
## Confidence level used: 0.95
## Intervals are back-transformed from the log scale
```

MODEL 2b - Does **depth** has an effect on **fish density m2** within **Scleractinia** between the two depth bands at Yongala?

GLM model

```
glm2b_sclera <- glmmTMB(RICHNESS_m2 ~ DEPTH_RANGE,
  family = Gamma(link = "log"), data = data_yong_sclera,
  REML = TRUE, na.action = na.omit
)
nrow(data_yong_sclera)

## [1] 17

glm2b_sclera %>% simulateResiduals(plot = TRUE)

## Warning in smooth.construct.tp.smooth.spec(object, dk$data, dk$knots):
basis dimension, k, increased to minimum possible
## Warning in smooth.construct.tp.smooth.spec(object, dk$data, dk$knots):
basis dimension, k, increased to minimum possible
## Warning in smooth.construct.tp.smooth.spec(object, dk$data, dk$knots):
basis dimension, k, increased to minimum possible
```

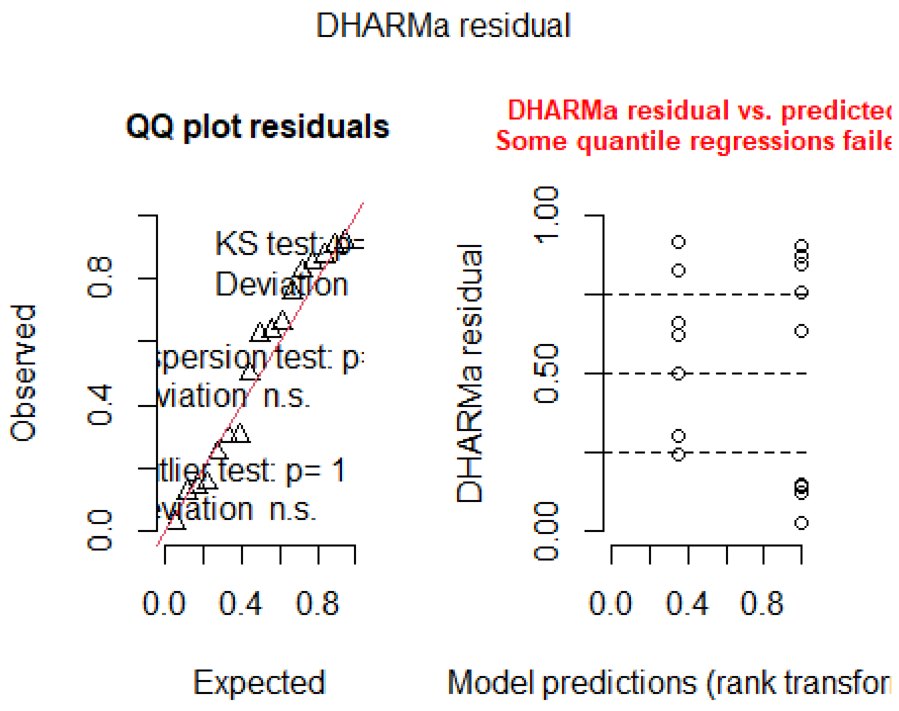


```
## Object of Class DHARMa with simulated residuals based on 250 simulations
with refit = FALSE . See ?DHARMa::simulateResiduals for help.
```

```
##
## Scaled residual values: 0.624 0.66 0.3 0.248 0.904 0.848 0.756 0.872 0.152
0.304 0.824 0.916 0.5 0.636 0.028 0.124 0.144

with(data_yong_sclera, leveneTest(log(RICHNESS_m2), DEPTH_RANGE))

## Levene's Test for Homogeneity of Variance (center = median)
##       Df F value  Pr(>F)
## group  1  3.7715 0.07115 .
##       15
## ---
## Signif. codes:  0 '***' 0.001 '**' 0.01 '*' 0.05 '.' 0.1 ' ' 1

# Significant differences in variation that may bias results towards
significance:
leveneTest(residuals(glm2b_sclera) ~ data_yong_sclera$DEPTH_RANGE)

## Levene's Test for Homogeneity of Variance (center = median)
##       Df F value   Pr(>F)
## group  1  15.381 0.001359 **
##       15
## ---
## Signif. codes:  0 '***' 0.001 '**' 0.01 '*' 0.05 '.' 0.1 ' ' 1
```

The above Levene's F-test on the model residuals highlights the unequal variances issue for this GLM. Therefore, we used a generalised least squres (GLS) approach that supports unequal variances.

GLS model

```
# Fit a GLS model, where variance changes between depth_range factors:
gls2b_sclera <- nlme::gls(
  log(RICHNESS_m2) ~ DEPTH_RANGE,
  data = data_yong_sclera,
  weights = varIdent(form = ~ 1 | DEPTH_RANGE)
)

qqnorm(gls2b_sclera$residuals)
qqline(gls2b_sclera$residuals) # not bad for normality
```

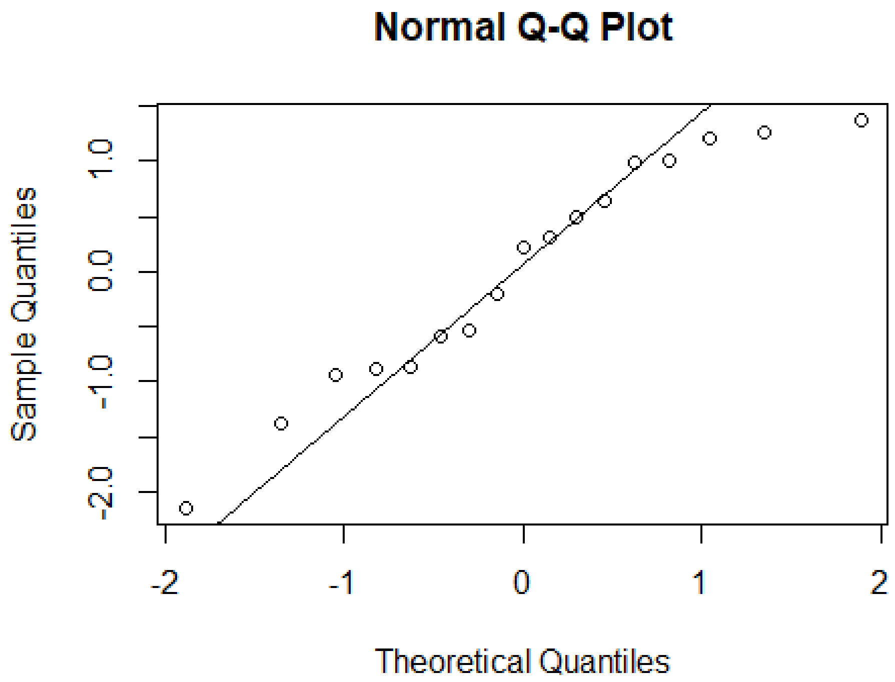


```
summary(gls2b_sclera)

## Generalized least squares fit by REML
##   Model: log(RICHNESS_m2) ~ DEPTH_RANGE
##   Data: data_yong_sclera
##        AIC      BIC    logLik
##   53.61067 56.44287 -22.80533
##
## Variance function:
##  Structure: Different standard deviations per stratum
##  Formula: ~1 | DEPTH_RANGE
##  Parameter estimates:
## 14 - 20 m 22 - 27 m
##  1.000000  2.036245
##
## Coefficients:
##                          Value Std.Error   t-value p-value
## (Intercept)          -3.1372736 0.2322092 -13.51055  0.0000
## DEPTH_RANGE22 - 27 m  0.4543766 0.5026455   0.90397  0.3803
##
##  Correlation:
##                      (Intr)
## DEPTH_RANGE22 - 27 m -0.462
##
## Standardized residuals:
##        Min         Q1        Med         Q3        Max
## -1.6053904 -0.8036478  0.3205518  0.8982438  1.5423848
```

```
##
## Residual standard error: 0.6567868
## Degrees of freedom: 17 total; 15 residual
```

```
gls2b_sclera %>% emmeans(~DEPTH_RANGE, type = "response", mode = "df.error")
```

```
##  DEPTH_RANGE response     SE df lower.CL upper.CL
##  14 - 20 m     0.0434 0.0101 14   0.0264   0.0714
##  22 - 27 m     0.0684 0.0305 14   0.0263   0.1779
##
## Degrees-of-freedom method: df.error
## Confidence level used: 0.95
## Intervals are back-transformed from the log scale
```

This model shows no evidence for richness differing by depth range within scleractinian corals.

MODEL 3 - Test for the effect of **coral taxa and site** on **fish density m2**

```
glmm3 <- glmmTMB(DENSITY_m2 ~ CORAL_ORDER * SITE + (1 | PAIR),
  family = Gamma(link = "log"), data = data_distinct,
  REML = TRUE, na.action = na.omit
)
nrow(data_distinct)
```

```
## [1] 46
```

```
glmm3 %>% simulateResiduals(plot = TRUE)
```

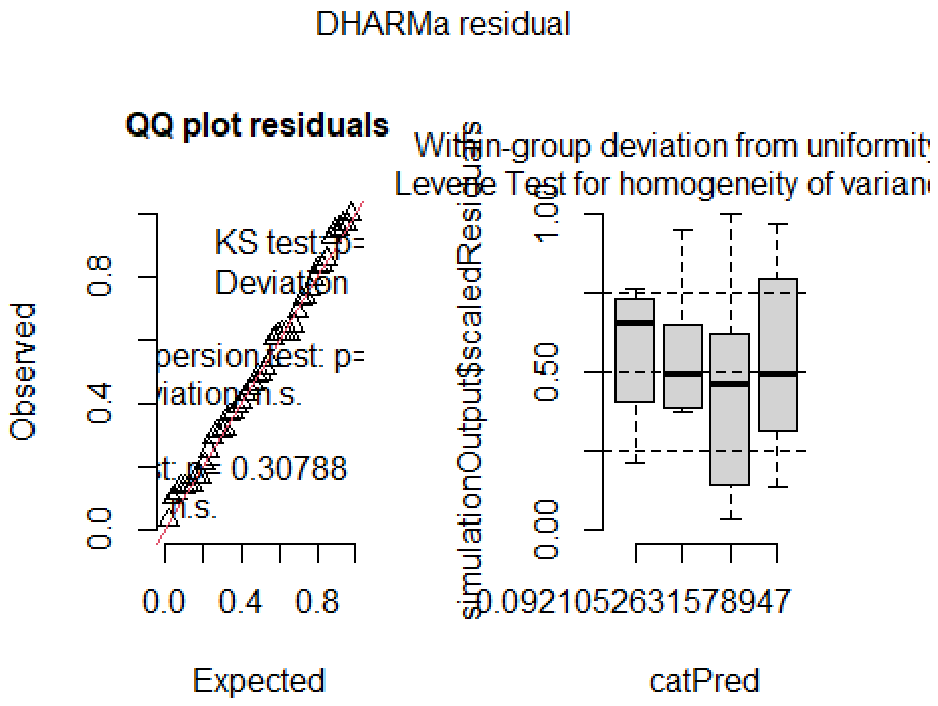


```
## Object of Class DHARMa with simulated residuals based on 250 simulations
with refit = FALSE . See ?DHARMa::simulateResiduals for help.
##
## Scaled residual values: 0.32 0.464 0.168 0.608 0.428 0.252 0.62 0.032
0.136 0.956 0.316 0.624 0.144 0.624 0.312 0.928 0.796 0.116 0.968 0.292 ...
```

```
Anova(glmm3)
```

```
## Analysis of Deviance Table (Type II Wald chisquare tests)
##
## Response: DENSITY_m2
##                      Chisq Df Pr(>Chisq)
## CORAL_ORDER        17.1184  1  3.512e-05 ***
## SITE               24.5958  1  7.071e-07 ***
## CORAL_ORDER:SITE    0.0263  1     0.8712
## ---
## Signif. codes:  0 '***' 0.001 '**' 0.01 '*' 0.05 '.' 0.1 ' ' 1
```

```
summary(glmm3)
```

```
##  Family: Gamma  ( log )
## Formula:          DENSITY_m2 ~ CORAL_ORDER * SITE + (1 | PAIR)
## Data: data_distinct
##
##      AIC      BIC   logLik deviance df.resid
##     83.1     94.1    -35.5     71.1       40
##
```

```
## Random effects:
##
## Conditional model:
##  Groups Name        Variance Std.Dev.
##  PAIR   (Intercept) 0.1217   0.3488
## Number of obs: 46, groups:  PAIR, 23
##
## Dispersion estimate for Gamma family (sigma^2): 0.909
##
## Conditional model:
##                                    Estimate Std. Error z value Pr(>|z|)
## (Intercept)                         -1.0519     0.4245  -2.478 0.013209 *
## CORAL_ORDERScleractinia             -1.3209     0.5659  -2.334 0.019595 *
## SITEYongala                          1.8073     0.4944   3.655 0.000257
***
## CORAL_ORDERScleractinia:SITEYongala   0.1091     0.6732   0.162 0.871245
## ---
## Signif. codes:  0 '***' 0.001 '**' 0.01 '*' 0.05 '.' 0.1 ' ' 1

# Marginal means and CIs:
emmeans::emmeans(glmm3, ~ CORAL_ORDER + SITE, type = "response")

##  CORAL_ORDER  SITE    response    SE  df asymp.LCL asymp.UCL
##  Antipatharia Orpheus   0.3493 0.148 Inf     0.152     0.803
##  Scleractinia Orpheus   0.0932 0.039 Inf     0.041     0.212
##  Antipatharia Yongala   2.1285 0.561 Inf     1.270     3.567
##  Scleractinia Yongala   0.6336 0.190 Inf     0.352     1.141
##
## Confidence level used: 0.95
## Intervals are back-transformed from the log scale

emmeans::emmeans(glmm3, revpairwise ~ SITE, type = "response")

## $emmeans
##  SITE    response     SE  df asymp.LCL asymp.UCL
##  Orpheus     0.18 0.0564 Inf    0.0978     0.333
##  Yongala     1.16 0.2540 Inf    0.7565     1.783
##
## Results are averaged over the levels of: CORAL_ORDER
## Confidence level used: 0.95
## Intervals are back-transformed from the log scale
##
## $contrasts
##  contrast          ratio   SE  df null z.ratio p.value
##  Yongala / Orpheus  6.44 2.41 Inf    1   4.962  <.0001
##
## Results are averaged over the levels of: CORAL_ORDER
## Tests are performed on the log scale

emmeans::emmeans(glmm3, pairwise ~ CORAL_ORDER, type = "response")
```

```
## $emmeans
##  CORAL_ORDER  response     SE  df asymp.LCL asymp.UCL
##  Antipatharia    0.862 0.2180 Inf     0.526     1.414
##  Scleractinia    0.243 0.0628 Inf     0.146     0.403
##
## Results are averaged over the levels of: SITE
## Confidence level used: 0.95
## Intervals are back-transformed from the log scale
##
## $contrasts
##  contrast                    ratio   SE  df null z.ratio p.value
##  Antipatharia / Scleractinia  3.55 1.18 Inf    1   3.806  0.0001
##
## Results are averaged over the levels of: SITE
## Tests are performed on the log scale
```

Prediction grid

```
dat_grid_3 <- with(
  data_distinct,
  expand.grid(
    CORAL_ORDER = unique(CORAL_ORDER),
    SITE = unique(SITE),
    AREA_m2 = mean(AREA_m2)
  )
)

dat_grid_3$DENSITY_m2 <-
  predict(glmm3, newdata = dat_grid_3, re.form = NA, type = "response")
dat_grid_3

##    CORAL_ORDER    SITE  AREA_m2 DENSITY_m2
## 1 Antipatharia Yongala 117.9022 2.31006807
## 2 Scleractinia Yongala 117.9022 0.74712896
## 3 Antipatharia Orpheus 117.9022 0.36338306
## 4 Scleractinia Orpheus 117.9022 0.09372772
```

MODEL 4 - Test for the effect of **coral taxa and site** on the **fish species richness**

```
glmm4 <- glmmTMB(RICHNESS_m2 ~ CORAL_ORDER * SITE + (1 | PAIR),
  family = Gamma(link = "log"), data = data_distinct,
  REML = TRUE, na.action = na.omit
)
nrow(data_distinct)

## [1] 46

glmm4 %>% simulateResiduals(plot = TRUE)
```

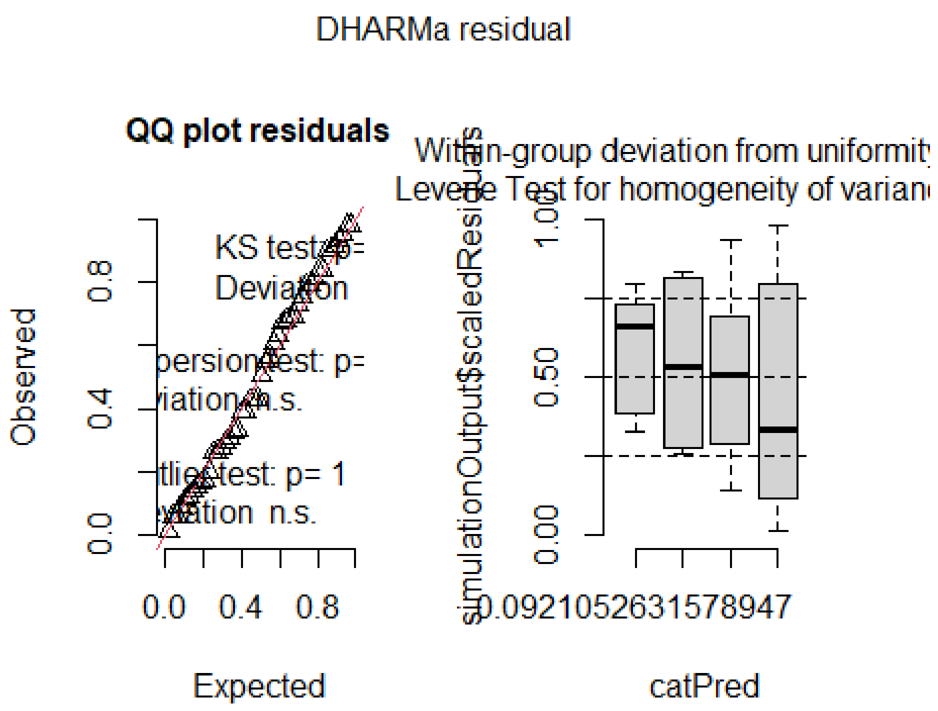


```
## Object of Class DHARMa with simulated residuals based on 250 simulations
with refit = FALSE . See ?DHARMa::simulateResiduals for help.
##
## Scaled residual values: 0.916 0.332 0.504 0.428 0.936 0.116 0.68 0.06
0.292 0.98 0.84 0.952 0.172 0.9 0.564 0.98 0.692 0.124 0.904 0.088 ...

Anova(glmm4)

## Analysis of Deviance Table (Type II Wald chisquare tests)
##
## Response: RICHNESS_m2
##                     Chisq Df Pr(>Chisq)
## CORAL_ORDER        0.0792  1     0.7784
## SITE              30.2954  1   3.71e-08 ***
## CORAL_ORDER:SITE   2.0776  1     0.1495
## ---
## Signif. codes:  0 '***' 0.001 '**' 0.01 '*' 0.05 '.' 0.1 ' ' 1

summary(glmm4)

##  Family: Gamma  ( log )
## Formula:          RICHNESS_m2 ~ CORAL_ORDER * SITE + (1 | PAIR)
## Data: data_distinct
##
##      AIC      BIC   logLik deviance df.resid
##   -167.2   -156.3     89.6   -179.2       40
##
```

```
## Random effects:
##
## Conditional model:
##  Groups Name        Variance  Std.Dev.
##  PAIR   (Intercept) 3.942e-09 6.279e-05
## Number of obs: 46, groups:  PAIR, 23
##
## Dispersion estimate for Gamma family (sigma^2): 0.548
##
## Conditional model:
##                                     Estimate Std. Error z value Pr(>|z|)
## (Intercept)                          -3.6619     0.3022 -12.118  < 2e-16
***
## CORAL_ORDERScleractinia              -0.4681     0.4273  -1.095  0.27331
## SITEYongala                           1.0097     0.3515   2.873  0.00407
**
## CORAL_ORDERScleractinia:SITEYongala   0.7165     0.4971   1.441  0.14948
## ---
## Signif. codes:  0 '***' 0.001 '**' 0.01 '*' 0.05 '.' 0.1 ' ' 1

emmeans::emmeans(glmm4, ~ CORAL_ORDER + SITE, type = "response")

##  CORAL_ORDER  SITE    response      SE  df asymp.LCL asymp.UCL
##  Antipatharia Orpheus   0.0257 0.00776 Inf    0.0142    0.0464
##  Scleractinia Orpheus   0.0161 0.00486 Inf    0.0089    0.0291
##  Antipatharia Yongala   0.0705 0.01270 Inf    0.0496    0.1002
##  Scleractinia Yongala   0.0904 0.01620 Inf    0.0636    0.1285
##
## Confidence level used: 0.95
## Intervals are back-transformed from the log scale

emmeans::emmeans(glmm4, revpairwise ~ SITE, type = "response")

## $emmeans
##  SITE    response      SE  df asymp.LCL asymp.UCL
##  Orpheus   0.0203 0.00434 Inf    0.0134    0.0309
##  Yongala   0.0798 0.01010 Inf    0.0622    0.1024
##
## Results are averaged over the levels of: CORAL_ORDER
## Confidence level used: 0.95
## Intervals are back-transformed from the log scale
##
## $contrasts
##  contrast          ratio    SE  df null z.ratio p.value
##  Yongala / Orpheus  3.93 0.976 Inf    1   5.504  <.0001
##
## Results are averaged over the levels of: CORAL_ORDER
## Tests are performed on the log scale
```

Prediction grid

```
dat_grid_4 <- with(
  data_distinct,
  expand.grid(
    CORAL_ORDER = unique(CORAL_ORDER),
    SITE = unique(SITE),
    AREA_m2 = mean(AREA_m2)
  )
)

dat_grid_4$DENSITY_m2 <-
  predict(glmm4, newdata = dat_grid_3, re.form = NA, type = "response")
dat_grid_4

##    CORAL_ORDER    SITE  AREA_m2 DENSITY_m2
## 1 Antipatharia Yongala 117.9022 0.07049875
## 2 Scleractinia Yongala 117.9022 0.09037060
## 3 Antipatharia Orpheus 117.9022 0.02568425
## 4 Scleractinia Orpheus 117.9022 0.01608270
```

FISH FUNCTIONAL DIVERSITY INDICES

MODEL 5 - Estimates of fish Functional Entities

Functional entities definition: Unique combinations of functional traits

```
data <- read_csv("../2_outputs/functional_analysis_data.csv", trim_ws = TRUE)
data$SITE <- as.factor(data$SITE)

library(gamlss)

## Warning: package 'gamlss' was built under R version 4.4.3

## Warning: package 'gamlss.dist' was built under R version 4.4.3

library(rcompanion)

## Warning: package 'rcompanion' was built under R version 4.4.3

plotNormalHistogram(data$nb_fe)
```

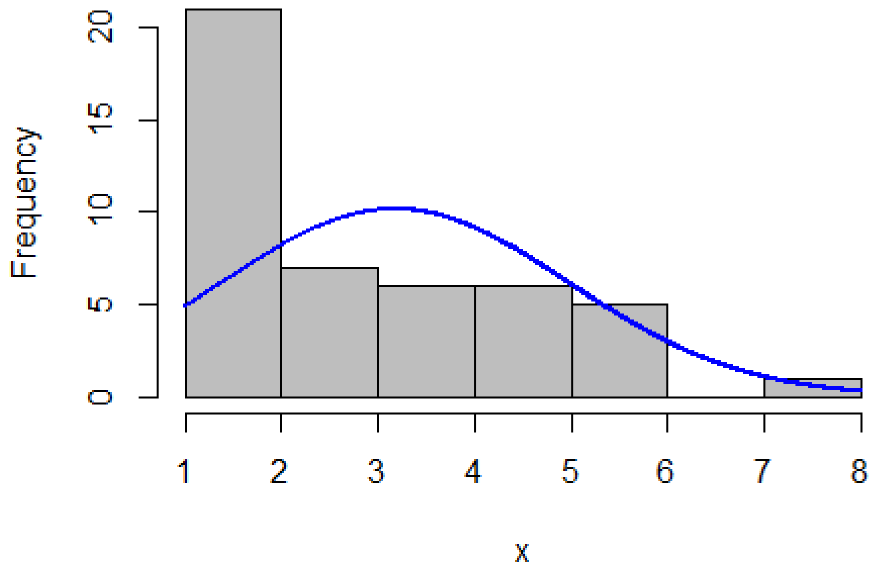


```
nb.fe.glmm <- glmmTMB(nb_fe ~ CORAL_ORDER + (1 | SITE), family = gaussian(),
data = data)

simulateResiduals(nb.fe.glmm, plot = TRUE)
```

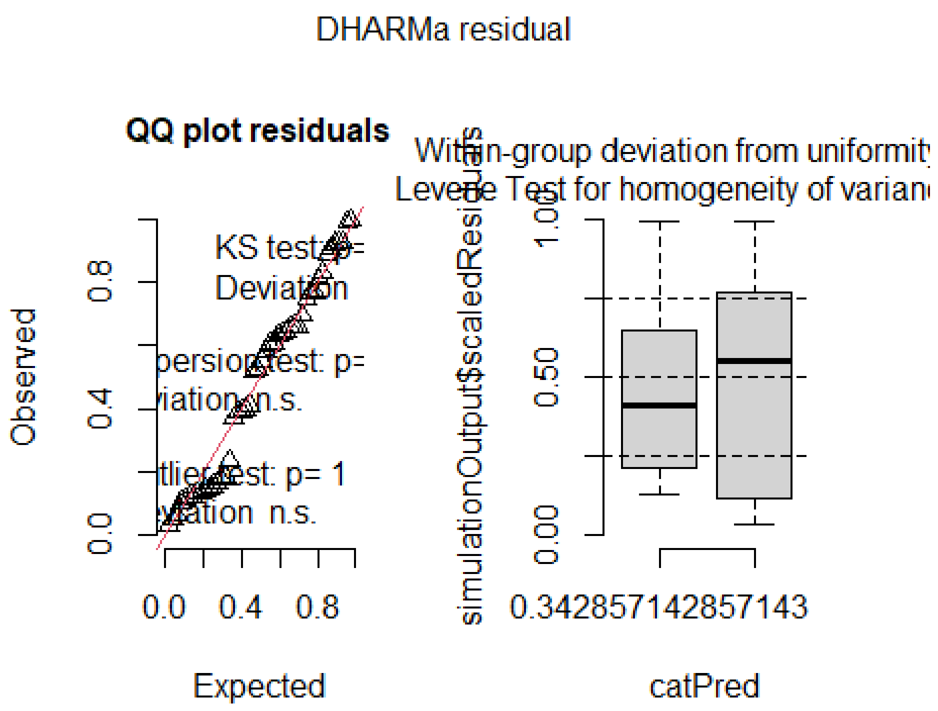


```
## Object of Class DHARMa with simulated residuals based on 250 simulations
with refit = FALSE . See ?DHARMa::simulateResiduals for help.
##
## Scaled residual values: 0.516 0.656 0.768 0.132 0.772 0.928 0.052 0.236
0.064 0.632 0.552 0.992 0.12 0.644 0.148 0.64 0.928 0.144 0.748 0.66 ...

shapiro.test(residuals(nb.fe.glmm))

##
##  Shapiro-Wilk normality test
##
## data:  residuals(nb.fe.glmm)
## W = 0.95693, p-value = 0.08707

nb.fe.glmm %>%
  emmeans(~CORAL_ORDER,
    type = "response"
  ) %>%
  as.data.frame()

##  CORAL_ORDER    emmean        SE df lower.CL upper.CL
##  Antipatharia 3.869567 0.3428278 42 3.177712 4.561421
##  Scleractinia 2.478259 0.3428278 42 1.786405 3.170114
##
## Confidence level used: 0.95

pairs(emmeans(nb.fe.glmm, ~CORAL_ORDER, type = "response"))
```

```
##   contrast                        estimate    SE df t.ratio p.value
##   Antipatharia - Scleractinia         1.39 0.485 42   2.870  0.0064
```

MODEL 6 - Estimates of fish Functional Redundancy

Functional Redundancy definition: Multiple species performing same or similar ecological roles

```
plotNormalHistogram(data$fred)
```

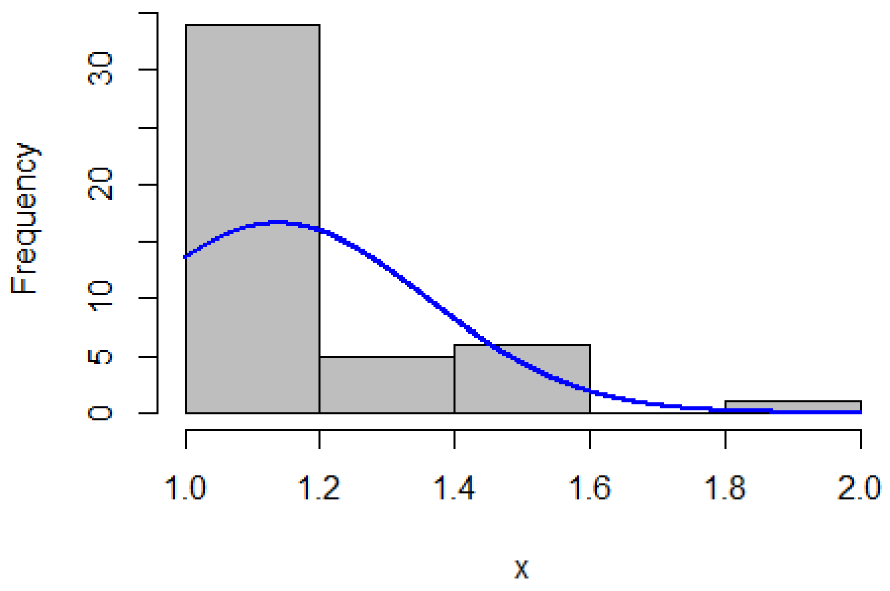


```
mean_y <- mean(data$fred)
var_y <- var(data$fred)
overdispersion_ratio <- var_y / mean_y
```

We have underdispersed and skewed data. We have decimal data; therefore, we use Generalized Additive Models for Location, Scale, and Shape (GAMLSS) which allow to model not just the mean (location) but also the dispersion (scale) and skewness/kurtosis (shape) of the data.

```
f.red.gamlss <- gamlss(fred ~ CORAL_ORDER, family = ZIPF(), data = data)

## GAMLSS-RS iteration 1: Global Deviance = 47.0145
## GAMLSS-RS iteration 2: Global Deviance = 47.0146

f.red.gamlss$converged

## [1] TRUE
```

```r
residuals_gamlss <- residuals(f.red.gamlss, type = "weighted")
hist(residuals_gamlss, main = "Histogram of Residuals", col = "lightblue")
```

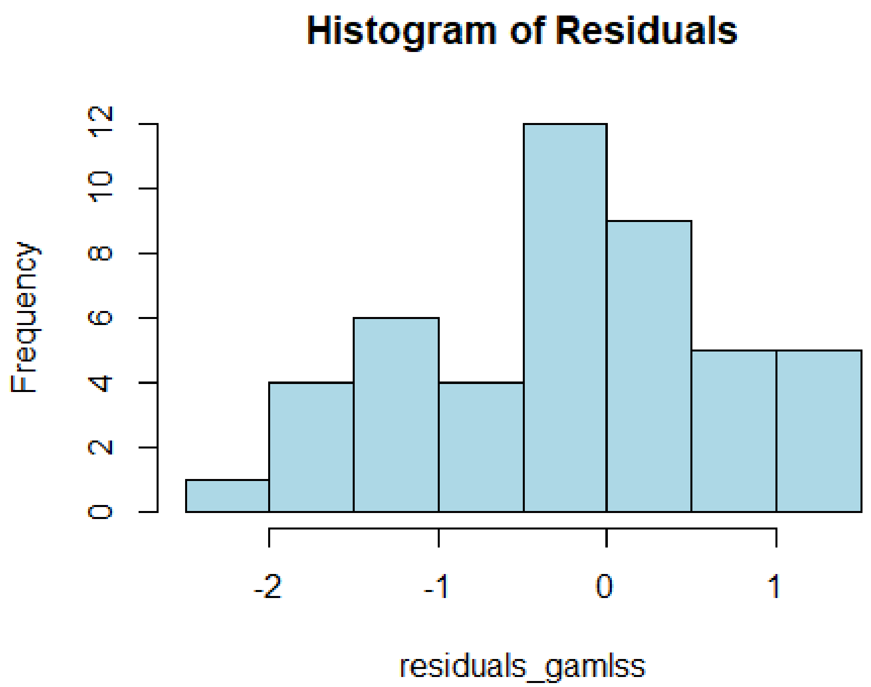


```r
plot(fitted(f.red.gamlss), residuals_gamlss,
  xlab = "Fitted values", ylab = "Residuals", main = "Residuals vs Fitted"
)
abline(h = 0, col = "red")
```

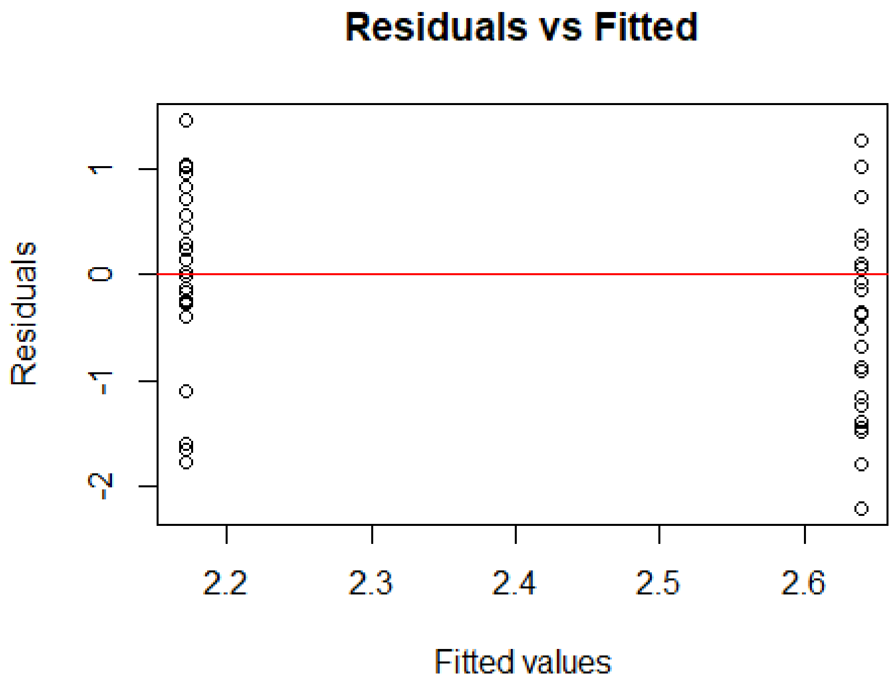


```
f.red.gamlss_GA <- gamlss(fred ~ CORAL_ORDER, family = GA(), data = data)

## GAMLSS-RS iteration 1: Global Deviance = -19.2936
## GAMLSS-RS iteration 2: Global Deviance = -19.2936

f.red.gamlss_GA$converged

## [1] TRUE

residuals_gamlss_GA <- residuals(f.red.gamlss_GA, type = "weighted")
hist(residuals_gamlss_GA, main = "Histogram of Residuals", col = "lightblue")
```

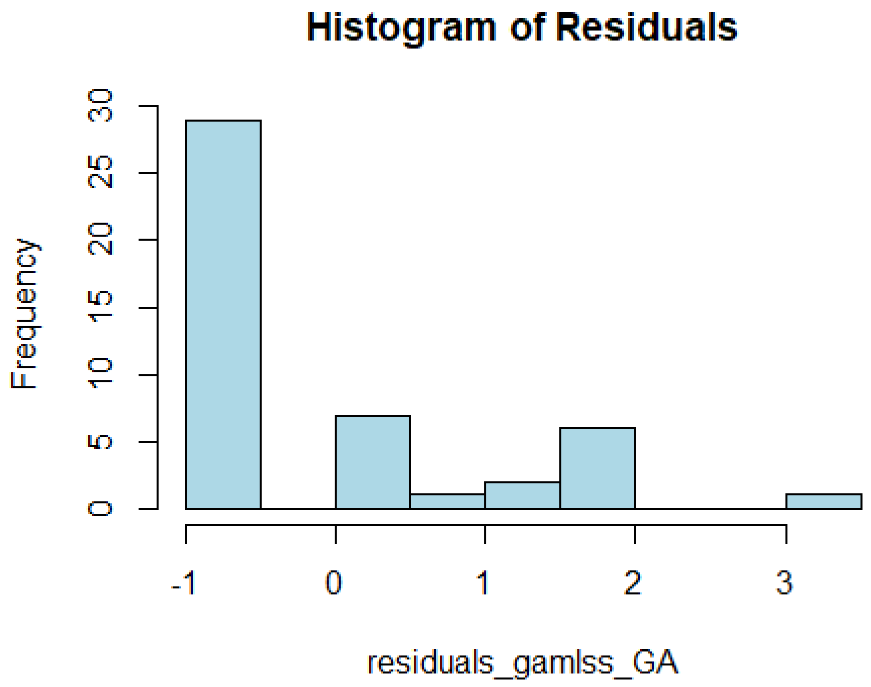


```r
plot(fitted(f.red.gamlss_GA), residuals_gamlss_GA,
  xlab = "Fitted values", ylab = "Residuals", main = "Residuals vs Fitted"
)
abline(h = 0, col = "red")
```

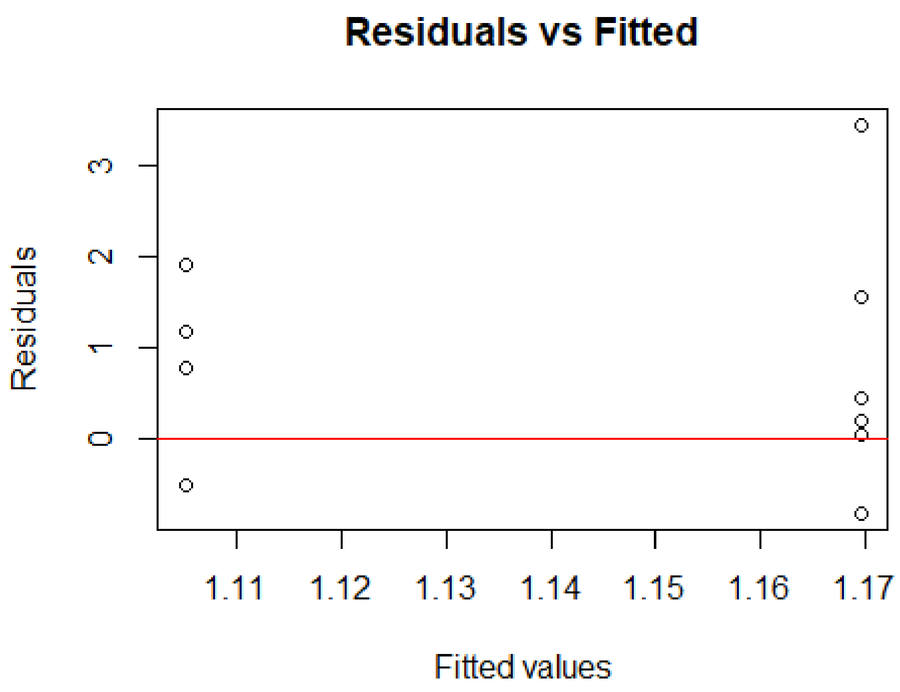


```
AIC(f.red.gamlss, f.red.gamlss_GA)

##                  df       AIC
## f.red.gamlss_GA   3 -13.29364
## f.red.gamlss      2  51.01461
```

Final Functional Redundancy Model without SITE

```
f.red.gamlss_GA %>%
  emmeans(~CORAL_ORDER,
    type = "response"
  ) %>%
  as.data.frame()

## ******************************************************************
## Family:  c("GA", "Gamma")
##
## Call:  gamlss(formula = fred ~ CORAL_ORDER, family = GA(),      data =
data)
##
## Fitting method: RS()
##
## ------------------------------------------------------------------
## Mu link function:  log
## Mu Coefficients:
##                            Estimate Std. Error t value Pr(>|t|)
## (Intercept)                 0.15663    0.03635   4.309 9.38e-05 ***
```

```
## CORAL_ORDERScleractinia -0.05672    0.05141  -1.103    0.276
## ---
## Signif. codes:  0 '***' 0.001 '**' 0.01 '*' 0.05 '.' 0.1 ' ' 1
##
## -------------------------------------------------------------------
## Sigma link function:  log
## Sigma Coefficients:
##             Estimate Std. Error t value Pr(>|t|)
## (Intercept)  -1.7467     0.1037  -16.84   <2e-16 ***
## ---
## Signif. codes:  0 '***' 0.001 '**' 0.01 '*' 0.05 '.' 0.1 ' ' 1
##
## -------------------------------------------------------------------
## No. of observations in the fit:  46
## Degrees of Freedom for the fit:  3
##       Residual Deg. of Freedom:  43
##                       at cycle:  2
##
## Global Deviance:     -19.29364
##             AIC:     -13.29364
##             SBC:     -7.807715
## *******************************************************************

##  CORAL_ORDER  response         SE df lower.CL upper.CL
##  Antipatharia 1.169565 0.04251794 43 1.086887 1.258532
##  Scleractinia 1.105072 0.04017339 43 1.026954 1.189134
##
## Confidence level used: 0.95
## Intervals are back-transformed from the log scale
```

```r
pairs(emmeans(f.red.gamlss_GA, ~CORAL_ORDER, type = "response"))
```

```
## *******************************************************************
## Family:  c("GA", "Gamma")
##
## Call:  gamlss(formula = fred ~ CORAL_ORDER, family = GA(),      data =
data)
##
## Fitting method: RS()
##
## -------------------------------------------------------------------
## Mu link function:  log
## Mu Coefficients:
##                         Estimate Std. Error t value Pr(>|t|)
## (Intercept)              0.15663    0.03635   4.309 9.38e-05 ***
## CORAL_ORDERScleractinia -0.05672    0.05141  -1.103    0.276
## ---
## Signif. codes:  0 '***' 0.001 '**' 0.01 '*' 0.05 '.' 0.1 ' ' 1
##
## -------------------------------------------------------------------
```

```
## Sigma link function:  log
## Sigma Coefficients:
##             Estimate Std. Error t value Pr(>|t|)
## (Intercept)  -1.7467     0.1037  -16.84   <2e-16 ***
## ---
## Signif. codes:  0 '***' 0.001 '**' 0.01 '*' 0.05 '.' 0.1 ' ' 1
##
## -------------------------------------------------------------------
## No. of observations in the fit:  46
## Degrees of Freedom for the fit:  3
##       Residual Deg. of Freedom:  43
##                       at cycle:  2
##
## Global Deviance:     -19.29364
##             AIC:     -13.29364
##             SBC:     -7.807715
## *******************************************************************

##  contrast                    ratio     SE df null t.ratio p.value
##  Antipatharia / Scleractinia  1.06 0.0544 43    1   1.103  0.2760
##
## Tests are performed on the log scale
```

MULTIVARIATE ANALYSIS

db-RDA

```
fish <- final_data %>% dplyr::select(U_ID, BINOMIAL, MAX_N)

env <- final_data %>% dplyr::select(U_ID, SITE, CORAL_MORPHO, AREA_m2,
CORAL_ORDER, SHELTER_dm3)
env2 <- distinct(env) %>%
  dplyr::select(-U_ID)



fish.mat <- fish %>%
  group_by(U_ID, BINOMIAL) %>%
  summarise(MAX_N = sum(MAX_N)) %>%
  ungroup()

fish.mat <- fish.mat %>%
  pivot_wider(names_from = BINOMIAL, values_from = MAX_N) %>%
  replace(is.na(.), 0)

fish.mat.2 <- fish.mat %>%
  dplyr::select(-U_ID)

fish.mat.2 <- as.matrix(fish.mat.2)
```

```r
fish.dist <- vegdist(wisconsin(sqrt(fish.mat.2)), method = "bray")

dbRDA <- dbrda(fish.dist ~ SITE + CORAL_ORDER + CORAL_MORPHO + SHELTER_dm3,
env2, dist = "bray")
summary(dbRDA)
```

```
##
## Call:
## dbrda(formula = fish.dist ~ SITE + CORAL_ORDER + CORAL_MORPHO +
SHELTER_dm3, data = env2, distance = "bray")
##
## Partitioning of squared Bray distance:
##               Inertia Proportion
## Total           17.08     1.0000
## Constrained      3.31     0.1938
## Unconstrained   13.77     0.8062
##
## Eigenvalues, and their contribution to the squared Bray distance
##
## Importance of components:
##                         dbRDA1  dbRDA2  dbRDA3  dbRDA4  dbRDA5   dbRDA6
dbRDA7
## Eigenvalue             1.31119 0.72694 0.63136 0.29801 0.22741 0.093040
0.02204
## Proportion Explained  0.07679 0.04257 0.03697 0.01745 0.01332 0.005449
0.00129
## Cumulative Proportion 0.07679 0.11936 0.15633 0.17379 0.18710 0.192553
0.19384
##                          MDS1   MDS2   MDS3    MDS4    MDS5    MDS6    MDS7
## Eigenvalue             2.6343 2.1874 1.8731 1.25369 1.09712 0.86889 0.73396
## Proportion Explained  0.1543 0.1281 0.1097 0.07342 0.06425 0.05089 0.04298
## Cumulative Proportion 0.3481 0.4762 0.5859 0.65933 0.72358 0.77447 0.81745
##                          MDS8    MDS9   MDS10   MDS11   MDS12   MDS13
MDS14
## Eigenvalue             0.67405 0.58425 0.49937 0.44945 0.41038 0.39226
0.3073
## Proportion Explained  0.03947 0.03422 0.02924 0.02632 0.02403 0.02297
0.0180
## Cumulative Proportion 0.85692 0.89114 0.92038 0.94670 0.97074 0.99371
1.0117
##                         MDS15    MDS16    MDS17    MDS18    MDS19    MDS20
## Eigenvalue             0.19646 0.170657 0.148036 0.115455 0.083417 0.076317
## Proportion Explained  0.01151 0.009994 0.008669 0.006761 0.004885 0.004469
## Cumulative Proportion 1.02321 1.033206 1.041876 1.048637 1.053522 1.057992
##                         MDS21    MDS22    MDS23    MDS24     MDS25
MDS26
## Eigenvalue             0.05020 0.043424 0.038833 0.026662 0.0068925
0.004559
## Proportion Explained  0.00294 0.002543 0.002274 0.001561 0.0004036
```

```
0.000267
## Cumulative Proportion 1.06093 1.063474 1.065748 1.067310 1.0677135
1.067980
##                            iMDS1     iMDS2     iMDS3     iMDS4     iMDS5
## Eigenvalue             -0.0151269 -0.019550 -0.027818 -0.036226 -0.065418
## Proportion Explained  -0.0008859 -0.001145 -0.001629 -0.002122 -0.003831
## Cumulative Proportion  1.0670946  1.065950  1.064321  1.062199  1.058368
##                          iMDS6    iMDS7     iMDS8     iMDS9    iMDS10
iMDS11
## Eigenvalue             -0.08571 -0.10160 -0.102892 -0.137913 -0.143131 -
0.167694
## Proportion Explained  -0.00502 -0.00595 -0.006026 -0.008077 -0.008382 -
0.009821
## Cumulative Proportion  1.05335  1.04740  1.041372  1.033296  1.024914
1.015093
##                         iMDS12
## Eigenvalue             -0.25772
## Proportion Explained  -0.01509
## Cumulative Proportion  1.00000
##
## Accumulated constrained eigenvalues
## Importance of components:
##                       dbRDA1 dbRDA2 dbRDA3  dbRDA4 dbRDA5  dbRDA6   dbRDA7
## Eigenvalue            1.3112 0.7269 0.6314 0.29801 0.2274 0.09304 0.022036
## Proportion Explained  0.3961 0.2196 0.1907 0.09003 0.0687 0.02811 0.006657
## Cumulative Proportion 0.3961 0.6158 0.8065 0.89653 0.9652 0.99334 1.000000
```

```
# Adding a constant to address negative eigenvalues
dbRDA_add <- dbrda(fish.dist ~ ., env2, dist = "bray", add = TRUE)

anova(dbRDA_add, by = "terms", perm.max = 500)
```

```
## Permutation test for dbrda under reduced model
## Terms added sequentially (first to last)
## Permutation: free
## Number of permutations: 999
##
## Model: dbrda(formula = fish.dist ~ SITE + CORAL_MORPHO + AREA_m2 +
CORAL_ORDER + SHELTER_dm3, data = env2, distance = "bray", add = TRUE)
##              Df SumOfSqs      F Pr(>F)
## SITE          1   0.9241 1.3575  0.079 .
## CORAL_MORPHO  4   3.4034 1.2499  0.014 *
## AREA_m2       1   0.7786 1.1437  0.232
## CORAL_ORDER   1   0.6142 0.9023  0.757
## SHELTER_dm3   1   0.7358 1.0808  0.309
## Residual     37  25.1873
## ---
## Signif. codes:  0 '***' 0.001 '**' 0.01 '*' 0.05 '.' 0.1 ' ' 1
```

```
anova(dbRDA_add, by = "axis", perm.max = 500) ## test axes for significance
```

```
## Permutation test for dbrda under reduced model
## Forward tests for axes
## Permutation: free
## Number of permutations: 999
##
## Model: dbrda(formula = fish.dist ~ SITE + CORAL_MORPHO + AREA_m2 +
CORAL_ORDER + SHELTER_dm3, data = env2, distance = "bray", add = TRUE)
##          Df SumOfSqs      F Pr(>F)
## dbRDA1    1   1.8269 2.6838  0.006 **
## dbRDA2    1   1.0995 1.6151  0.704
## dbRDA3    1   1.0507 1.5434  0.704
## dbRDA4    1   0.6789 0.9973  1.000
## dbRDA5    1   0.6003 0.8819
## dbRDA6    1   0.4688 0.6886
## dbRDA7    1   0.4068 0.5976
## dbRDA8    1   0.3241 0.4761
## Residual 37  25.1873
## ---
## Signif. codes:  0 '***' 0.001 '**' 0.01 '*' 0.05 '.' 0.1 ' ' 1
```

```
anova(dbRDA)
```

```
## Permutation test for dbrda under reduced model
## Permutation: free
## Number of permutations: 999
##
## Model: dbrda(formula = fish.dist ~ SITE + CORAL_ORDER + CORAL_MORPHO +
SHELTER_dm3, data = env2, distance = "bray")
##          Df SumOfSqs      F Pr(>F)
## Model     7    3.310 1.3053  0.032 *
## Residual 38   13.766
## ---
## Signif. codes:  0 '***' 0.001 '**' 0.01 '*' 0.05 '.' 0.1 ' ' 1
```

The overall test of the significance of the analysis was found significant

R2 Marginal and Conditional

```
adjR2.rda <- RsquareAdj(dbRDA)$adj.r.squared
adjR2.rda
```

```
## [1] 0.04534073
```

```
ef <- envfit(dbRDA, env2, permutations = 0)
ef
```

```
##
## ***VECTORS
##
##                 dbRDA1   dbRDA2     r2
## AREA_m2        0.99482 -0.10161 0.0826
## SHELTER_dm3    0.94841 -0.31703 0.0716
```

```
##
## ***FACTORS:
##
## Centroids:
##                           dbRDA1  dbRDA2
## SITEOrpheus               0.3621  0.8439
## SITEYongala              -0.1278 -0.2979
## CORAL_MORPHObranching     0.6441 -0.0647
## CORAL_MORPHOencrusting   -1.8160  0.7363
## CORAL_MORPHOfoliose      -0.6692 -0.8742
## CORAL_MORPHOmassive      -0.7351  0.2602
## CORAL_MORPHOtabular       0.7979  0.1516
## CORAL_ORDERAntipatharia   0.6904 -0.1579
## CORAL_ORDERScleractinia  -0.6904  0.1579
##
## Goodness of fit:
##                  r2
## SITE         0.0927
## CORAL_MORPHO 0.1972
## CORAL_ORDER  0.1562
```

Significant Variables

```
anova(dbRDA, by = "terms", perm.max = 500)
```

```
## Permutation test for dbrda under reduced model
## Terms added sequentially (first to last)
## Permutation: free
## Number of permutations: 999
##
## Model: dbrda(formula = fish.dist ~ SITE + CORAL_ORDER + CORAL_MORPHO +
SHELTER_dm3, data = env2, distance = "bray")
##              Df SumOfSqs      F Pr(>F)
## SITE          1   0.6004 1.6574  0.083 .
## CORAL_ORDER   1   1.1327 3.1267  0.001 ***
## CORAL_MORPHO  4   1.2664 0.8739  0.777
## SHELTER_dm3   1   0.3106 0.8574  0.599
## Residual     38  13.7656
## ---
## Signif. codes:  0 '***' 0.001 '**' 0.01 '*' 0.05 '.' 0.1 ' ' 1
```

Coral taxa was found significant

one-way PERMANOVA - Coral Taxa

```
set.seed(36)

fish.coral <- adonis2(fish.dist ~ CORAL_ORDER, data = env2, permutations =
999, method = "bray")
fish.coral
```

```
## Permutation test for adonis under reduced model
## Permutation: free
## Number of permutations: 999
##
## adonis2(formula = fish.dist ~ CORAL_ORDER, data = env2, permutations =
999, method = "bray")
##          Df SumOfSqs      R2      F Pr(>F)
## Model     1   1.1327 0.06633 3.1259  0.001 ***
## Residual 44  15.9430 0.93367
## Total    45  17.0756 1.00000
## ---
## Signif. codes:  0 '***' 0.001 '**' 0.01 '*' 0.05 '.' 0.1 ' ' 1
```

Test the primary assumption of PERMANOVA - that the observations are independent and they have similar distributions

```
dispersion <- betadisper(fish.dist, group = env2$CORAL_ORDER)

plot(dispersion)
```

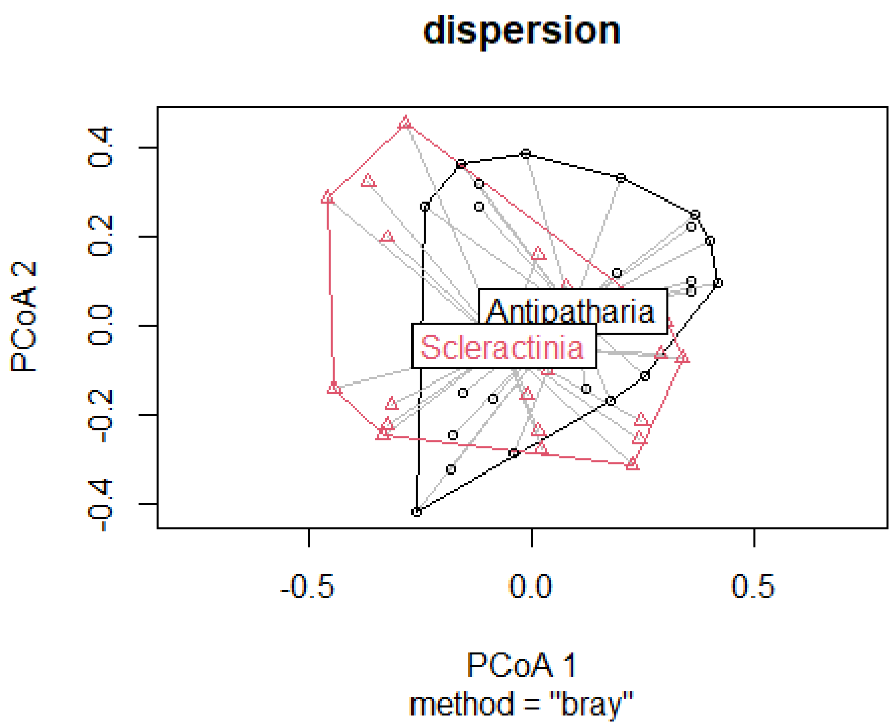


```
permutest(dispersion)

##
## Permutation test for homogeneity of multivariate dispersions
## Permutation: free
## Number of permutations: 999
##
```

```
## Response: Distances
##           Df  Sum Sq  Mean Sq      F N.Perm Pr(>F)
## Groups     1 0.01218 0.012178 0.9782    999  0.362
## Residuals 44 0.54774 0.012449
```

```
anova(dispersion)
```

```
## Analysis of Variance Table
##
## Response: Distances
##           Df  Sum Sq  Mean Sq F value Pr(>F)
## Groups     1 0.01218 0.012178  0.9782  0.328
## Residuals 44 0.54774 0.012449
```

```
TukeyHSD(dispersion)
```

```
##   Tukey multiple comparisons of means
##     95% family-wise confidence level
##
## Fit: aov(formula = distances ~ group, data = df)
##
## $group
##                                   diff         lwr        upr     p adj
## Scleractinia-Antipatharia 0.03254141 -0.03376671 0.09884954 0.3280404
```

```
boxplot(dispersion, xlab = "", las = 2, cex.axis = 0.8)
```

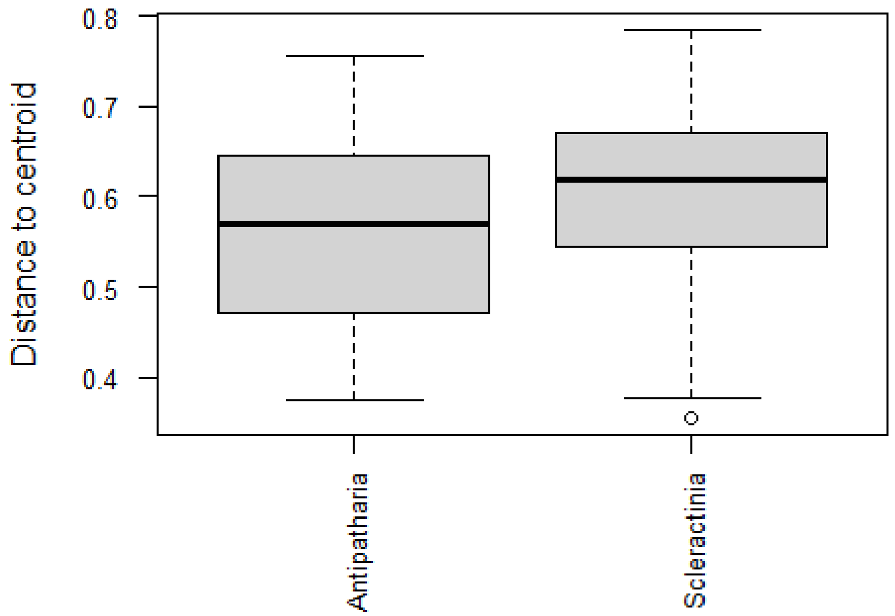

Equal dispersion within distinct coral taxa

AICc MODEL SELECTION

Model 1

```
glmm1_dxc <- glmmTMB(DENSITY_m2 ~ DEPTH_RANGE * CORAL_ORDER + (1 | PAIR),
  family = Gamma(link = "log"), data = data_yong,
  REML = TRUE, na.action = na.omit
)
glmm1_dc <- glmmTMB(DENSITY_m2 ~ DEPTH_RANGE + CORAL_ORDER + (1 | PAIR),
  family = Gamma(link = "log"), data = data_yong,
  REML = TRUE, na.action = na.omit
)
glmm1_d <- glmmTMB(DENSITY_m2 ~ DEPTH_RANGE + (1 | PAIR),
  family = Gamma(link = "log"), data = data_yong,
  REML = TRUE, na.action = na.omit
)
glmm1_c <- glmmTMB(DENSITY_m2 ~ CORAL_ORDER + (1 | PAIR),
  family = Gamma(link = "log"), data = data_yong,
  REML = TRUE, na.action = na.omit
)
glmm1_null <- glmmTMB(DENSITY_m2 ~ 1 + (1 | PAIR),
  family = Gamma(link = "log"), data = data_yong,
  REML = TRUE, na.action = na.omit
)

best_model_1 <- MuMIn::AICc(glmm1_dxc, glmm1_dc, glmm1_d, glmm1_c,
glmm1_null) %>%
  arrange(AICc) %>%
  mutate("ΔAICc" = AICc - AICc[1])
```

The best fit model (lowest AICc) for question 1 is one with CORAL_ORDER only, and no other model is within 2 AICc.

Model 2

```
# Fit model:
glmm2_dxc <- glmmTMB(RICHNESS_m2 ~ DEPTH_RANGE * CORAL_ORDER + (1 | PAIR),
  family = Gamma(link = "log"), data = data_yong,
  REML = TRUE, na.action = na.omit
)
glmm2_dc <- glmmTMB(RICHNESS_m2 ~ DEPTH_RANGE + CORAL_ORDER + (1 | PAIR),
  family = Gamma(link = "log"), data = data_yong,
  REML = TRUE, na.action = na.omit
)
glmm2_d <- glmmTMB(RICHNESS_m2 ~ DEPTH_RANGE + (1 | PAIR),
  family = Gamma(link = "log"), data = data_yong,
  REML = TRUE, na.action = na.omit
)
glmm2_c <- glmmTMB(RICHNESS_m2 ~ CORAL_ORDER + (1 | PAIR),
  family = Gamma(link = "log"), data = data_yong,
```

```
  REML = TRUE, na.action = na.omit
)
glmm2_null <- glmmTMB(RICHNESS_m2 ~ 1 + (1 | PAIR),
  family = Gamma(link = "log"), data = data_yong,
  REML = TRUE, na.action = na.omit
)

best_model_2 <- MuMIn::AICc(glmm2_dxc, glmm2_dc, glmm2_d, glmm2_c,
glmm2_null) %>%
  arrange(AICc) %>%
  mutate("ΔAICc" = AICc - AICc[1])
```

The best fit model (lowest AICc) for question 2 is the null model (no variables explain species richness well) with DEPTH_RANGE × CORAL_ORDER (full interaction model) being suboptimal but within 2 AICc so should also be considered.

Model 3

```
glmm3_cxs <- glmmTMB(DENSITY_m2 ~ CORAL_ORDER * SITE + (1 | PAIR),
  family = Gamma(link = "log"), data = data_distinct,
  REML = TRUE, na.action = na.omit
)
glmm3_cs <- glmmTMB(DENSITY_m2 ~ CORAL_ORDER + SITE + (1 | PAIR),
  family = Gamma(link = "log"), data = data_distinct,
  REML = TRUE, na.action = na.omit
)
glmm3_c <- glmmTMB(DENSITY_m2 ~ CORAL_ORDER + (1 | PAIR),
  family = Gamma(link = "log"), data = data_distinct,
  REML = TRUE, na.action = na.omit
)
glmm3_s <- glmmTMB(DENSITY_m2 ~ SITE + (1 | PAIR),
  family = Gamma(link = "log"), data = data_distinct,
  REML = TRUE, na.action = na.omit
)
glmm3_null <- glmmTMB(DENSITY_m2 ~ 1 + (1 | PAIR),
  family = Gamma(link = "log"), data = data_distinct,
  REML = TRUE, na.action = na.omit
)

best_model_3 <- MuMIn::AICc(glmm3_cxs, glmm3_cs, glmm3_c, glmm3_s,
glmm3_null) %>%
  arrange(AICc) %>%
  mutate("ΔAICc" = AICc - AICc[1])
```

The best fit model (lowest AICc) for question 3 is one with CORAL_ORDER and SITE (additively), but the suboptimal model within 2 AICc includes the full interaction of CORAL_ORDER × SITE.

Model 4

```
glmm4_cxs <- glmmTMB(RICHNESS_m2 ~ CORAL_ORDER * SITE + (1 | PAIR),
  family = Gamma(link = "log"), data = data_distinct,
  REML = TRUE, na.action = na.omit
)
glmm4_cs <- glmmTMB(RICHNESS_m2 ~ CORAL_ORDER + SITE + (1 | PAIR),
  family = Gamma(link = "log"), data = data_distinct,
  REML = TRUE, na.action = na.omit
)
glmm4_c <- glmmTMB(RICHNESS_m2 ~ CORAL_ORDER + (1 | PAIR),
  family = Gamma(link = "log"), data = data_distinct,
  REML = TRUE, na.action = na.omit
)
glmm4_s <- glmmTMB(RICHNESS_m2 ~ SITE + (1 | PAIR),
  family = Gamma(link = "log"), data = data_distinct,
  REML = TRUE, na.action = na.omit
)
glmm4_null <- glmmTMB(RICHNESS_m2 ~ 1 + (1 | PAIR),
  family = Gamma(link = "log"), data = data_distinct,
  REML = TRUE, na.action = na.omit
)

best_model_4 <- MuMIn::AICc(glmm4_cxs, glmm4_cs, glmm4_c, glmm4_s,
glmm4_null) %>%
  arrange(AICc) %>%
  mutate("ΔAICc" = AICc - AICc[1])
```

The best fit model (lowest AICc) for question 4 is one with SITE only, with no other model within 2 AICc.
