# Supplementary material for: Neglected Biodiversity of Fish Assemblages Associated With Antipatharia (Black Corals) on Tropical Shallow Reef Ecosystems
Source: Ecol Evol. 2025 Aug 21;15(8):e72015. doi: 10.1002/ece3.72015 (PMC12370845; doi:10.1002/ece3.72015)
Supplement: Supplementary file 3 — Appendix S3: ece372015‐sup‐0003‐AppendixS3.pdf. [file ECE3-15-e72015-s002.pdf]

Supplementary 3

This script shows the functional trait space analysis for Gress et al. We used the package mFD and followed material provided by the package authors from this tutorial: https://cran.r-project.org/web/packages/mFD/vignettes/mFD_general_workflow.html

The basic steps are:

1. Compute a pairwise trait-based distance matrix using the species x traits df.
2. Perform a PCoA with this trait based distance matrix
3. Evaluate the quality of PCoA-based multidimensional spaces
4. Test correlation between functional axes and traits
5. Plot trait space

Data

Our data are available at the following link: https://figshare.com/s/f0f6d8fc855a81628b66

```
data <- read_csv("../2_outputs/data_with_traits.csv", trim_ws = TRUE)
```

Examine Traits

fish_traits_subset

Size	Mobility	Activity_text	Schooling	Position	Diet_Mouillot_text
2	1	diurnal	2	1	invertivore-mobile
3	2	diurnal	3	1	invertivore-mobile
2	1	diurnal	4	2	planktivore
2	1	diurnal	5	2	planktivore
2	1	diurnal	5	2	planktivore
2	1	diurnal	3	1	planktivore
5	3	diurnal	4	2	omnivore
4	2	diurnal	1	1	invertivore-mobile
6	2	diurnal	3	1	omnivore
4	1	day/night	1	1	piscivore
4	1	day/night	1	1	piscivore
4	2	nocturnal	4	2	piscivore
1	1	nocturnal	4	2	planktivore
1	2	nocturnal	4	1	invertivore-mobile
1	2	nocturnal	5	2	planktivore
3	3	diurnal	5	3	planktivore
1	1	nocturnal	5	2	planktivore
3	1	diurnal	2	1	omnivore
2	1	diurnal	5	2	planktivore
4	2	nocturnal	4	2	piscivore
6	2	day/night	1	1	piscivore
1	1	nocturnal	4	2	planktivore

4	2	nocturnal	3	2	piscivore
6	2	diurnal	1	1	piscivore
3	2	diurnal	3	1	invertivore-mobile
2	1	nocturnal	3	2	piscivore
1	1	nocturnal	4	2	planktivore
4	3	diurnal	4	2	omnivore
6	2	diurnal	4	1	invertivore-mobile
2	2	diurnal	1	1	invertivore-mobile
4	1	diurnal	1	1	invertivore-sessile
4	1	nocturnal	1	2	piscivore
2	1	diurnal	2	1	invertivore-sessile
3	1	diurnal	2	1	invertivore-mobile
2	1	diurnal	3	1	omnivore
3	1	diurnal	2	1	invertivore-sessile
2	1	diurnal	4	1	omnivore

Data Preparation

Construct a species x trait matrix

```
all.fish.traits <- data %>% select(BINOMIAL, `Trophic Level`, `Trophic Group`, `Functional Group`,

all.fish.traits <- all.fish.traits %>% mutate(across(c(3:17), as.factor))

# taken a sub set following the Parravicini 2020 and Mouillot 2014 papers - many of the traits in
fish.traits.sub <-all.fish.traits %>% select(BINOMIAL, Size, Mobility, Activity_text,Schooling,Po

# Define trait types
species.traits <- fish.traits.sub %>% mutate(
  Size = as.ordered(Size),
  Mobility = as.ordered(Mobility),
  Activity_text = as.factor(Activity_text),
  Schooling = as.ordered(Schooling),
  Position = as.ordered(Position),
```

```
  Diet_Mouillot_text = as.factor(Diet_Mouillot_text)
)

# BINOMIAL must be the rowname
species.traits <- species.traits %>% column_to_rownames(var="BINOMIAL")
```

species x assemblage matrix

```
species.assemblage <- data %>% select(U_ID, BINOMIAL, MAX_N)

# Pivot to wide format
species.assemblage <- species.assemblage %>%  pivot_wider(names_from = BINOMIAL,
                                    values_from = MAX_N,
                                    values_fn = list(MAX_N = sum),
                                    values_fill = 0)

# U_ID must be the rowname
species.assemblage <- species.assemblage %>% column_to_rownames(var="U_ID")
```

trait definitions - tells the mFD package how to treat these taits as some are ordinal etc...

```
# Define traits

trait.definitions <- data.frame(
  trait_name = c("Size", "Mobility", "Activity_text", "Schooling", "Position", "Diet_Mouillot_tex
  trait_type = c("O", "O", "N", "O", "O", "N")
                            )

# Now summarise the trait data using mFD
# Species traits summary:
trait.summary <- mFD::sp.tr.summary(
  tr_cat     = trait.definitions,   # the definitions
  sp_tr      = species.traits, # the species traits we have
  stop_if_NA = TRUE)

trait.summary$"tr_types"
```

```
$Size
[1] "ordered" "factor"

$Mobility
[1] "ordered" "factor"

$Activity_text
[1] "factor"

$Schooling
[1] "ordered" "factor"
```

```
$Position
[1] "ordered" "factor"

$Diet_Mouillot_text
[1] "factor"
```

```
trait.summary$"mod_list"
```

```
$Size
[1] 2 3 5 4 6 1
Levels: 1 < 2 < 3 < 4 < 5 < 6

$Mobility
[1] 1 2 3
Levels: 1 < 2 < 3

$Activity_text
[1] diurnal   day/night nocturnal
Levels: day/night diurnal nocturnal

$Schooling
[1] 2 3 4 5 1
Levels: 1 < 2 < 3 < 4 < 5

$Position
[1] 1 2 3
Levels: 1 < 2 < 3

$Diet_Mouillot_text
[1] invertivore-mobile  planktivore         omnivore
[4] piscivore           invertivore-sessile
5 Levels: invertivore-mobile invertivore-sessile omnivore ... planktivore
```

Step 1 - Compute a trait-based distance matrix

To compute trait-based distances, we will use the mFD::funct.dist() function

```
sp_dist_matrix <- mFD::funct.dist(
  sp_tr         = species.traits, # our species traits data frame
  tr_cat        = trait.definitions, # the definitions summarising the traits
  metric        = "gower", # type of distance metric - if you have non-continuous traits use the (
  scale_euclid  = "scale_center",
  ordinal_var   = "classic",
  weight_type   = "equal",
  stop_if_NA    = TRUE)
```

```
Warning in mFD::funct.dist(sp_tr = species.traits, tr_cat = trait.definitions,
: Functional distance between some species is equal to 0. You can choose to
gather species into Functional Entities gathering species with similar traits
values.
```

Compute functional entities

```
sp_to_fe <- mFD::sp.to.fe(
  sp_tr       = species.traits,
  tr_cat      = trait.definitions,
  fe_nm_type  = "fe_rank",
  check_input = TRUE)

# FE version of the species.trait matrix
species.traits.FE <-sp_to_fe$fe_tr
```

Repeat distance matrix calculation now using Functional entities

```
sp_dist_matrix <- mFD::funct.dist(
  sp_tr         = species.traits.FE, # our FE species traits data frame
  tr_cat        = trait.definitions, # the definitions summarising the traits
  metric        = "gower", # type of distance metric - if you have non-continuous traits use the (
  scale_euclid  = "scale_center",
  ordinal_var   = "classic",
  weight_type   = "equal",
  stop_if_NA    = TRUE)

round(sp_dist_matrix, 3)              # Output of the function mFD::funct.dist()
```

```
      fe_1  fe_2  fe_3  fe_4  fe_5  fe_6  fe_7  fe_8  fe_9 fe_10 fe_11 fe_12
fe_2  0.242
fe_3  0.450 0.608
fe_4  0.650 0.642 0.533
fe_5  0.525 0.350 0.492 0.458
fe_6  0.375 0.533 0.158 0.442 0.650
fe_7  0.042 0.200 0.408 0.608 0.483 0.333
fe_8  0.167 0.325 0.283 0.483 0.608 0.208 0.125
fe_9  0.475 0.633 0.442 0.742 0.450 0.600 0.433 0.558
fe_10 0.567 0.725 0.117 0.417 0.542 0.192 0.525 0.400 0.492
fe_11 0.550 0.708 0.267 0.567 0.525 0.425 0.508 0.383 0.242 0.317
fe_12 0.575 0.333 0.275 0.642 0.350 0.367 0.533 0.492 0.633 0.392 0.542
fe_13 0.283 0.125 0.567 0.767 0.308 0.658 0.325 0.450 0.592 0.683 0.667 0.292
fe_14 0.283 0.525 0.500 0.867 0.575 0.658 0.325 0.450 0.358 0.617 0.600 0.692
fe_15 0.200 0.042 0.650 0.683 0.392 0.575 0.242 0.367 0.675 0.767 0.750 0.375
fe_16 0.408 0.567 0.292 0.408 0.617 0.200 0.367 0.242 0.400 0.325 0.225 0.567
fe_17 0.800 0.792 0.517 0.150 0.442 0.592 0.758 0.633 0.658 0.400 0.417 0.625
fe_18 0.567 0.392 0.450 0.417 0.042 0.608 0.525 0.567 0.492 0.500 0.483 0.392
fe_19 0.633 0.792 0.350 0.317 0.442 0.425 0.592 0.467 0.492 0.233 0.250 0.625
fe_20 0.417 0.242 0.533 0.400 0.192 0.458 0.375 0.417 0.642 0.650 0.633 0.408
```

```
fe_21 0.442 0.600 0.408 0.708 0.417 0.567 0.400 0.525 0.033 0.458 0.275 0.600
fe_22 0.508 0.667 0.142 0.608 0.483 0.300 0.467 0.425 0.367 0.192 0.208 0.333
fe_23 0.500 0.658 0.117 0.483 0.608 0.125 0.458 0.333 0.558 0.067 0.383 0.325
fe_24 0.483 0.642 0.367 0.333 0.625 0.275 0.442 0.317 0.575 0.250 0.400 0.642
fe_25 0.567 0.392 0.617 0.250 0.208 0.525 0.525 0.567 0.658 0.500 0.650 0.558
fe_26 0.375 0.533 0.325 0.442 0.650 0.167 0.333 0.208 0.600 0.358 0.425 0.533
fe_27 0.408 0.567 0.125 0.408 0.617 0.033 0.367 0.242 0.567 0.158 0.392 0.400
fe_28 0.333 0.492 0.283 0.483 0.608 0.208 0.292 0.167 0.392 0.400 0.217 0.492
fe_29 0.408 0.567 0.292 0.408 0.617 0.200 0.367 0.242 0.567 0.325 0.392 0.567
fe_30 0.292 0.450 0.325 0.525 0.567 0.250 0.250 0.208 0.350 0.442 0.258 0.450
      fe_13 fe_14 fe_15 fe_16 fe_17 fe_18 fe_19 fe_20 fe_21 fe_22 fe_23 fe_24
fe_2
fe_3
fe_4
fe_5
fe_6
fe_7
fe_8
fe_9
fe_10
fe_11
fe_12
fe_13
fe_14 0.400
fe_15 0.083 0.483
fe_16 0.692 0.625 0.608
fe_17 0.750 0.850 0.833 0.558
fe_18 0.350 0.617 0.433 0.575 0.400
fe_19 0.750 0.683 0.833 0.392 0.167 0.400
fe_20 0.367 0.700 0.283 0.492 0.550 0.150 0.550
fe_21 0.558 0.325 0.642 0.367 0.692 0.458 0.525 0.608
fe_22 0.625 0.558 0.708 0.433 0.458 0.525 0.292 0.675 0.400
fe_23 0.617 0.617 0.700 0.325 0.467 0.567 0.300 0.583 0.525 0.258
fe_24 0.767 0.700 0.683 0.242 0.483 0.583 0.317 0.567 0.542 0.442 0.317
fe_25 0.517 0.783 0.433 0.492 0.400 0.167 0.400 0.150 0.625 0.692 0.567 0.417
fe_26 0.658 0.658 0.575 0.200 0.592 0.608 0.425 0.458 0.567 0.467 0.292 0.108
fe_27 0.692 0.625 0.608 0.167 0.558 0.575 0.392 0.492 0.533 0.267 0.158 0.242
fe_28 0.617 0.617 0.533 0.075 0.633 0.567 0.467 0.417 0.358 0.425 0.333 0.317
fe_29 0.692 0.625 0.608 0.167 0.558 0.575 0.392 0.492 0.533 0.433 0.325 0.075
fe_30 0.575 0.575 0.492 0.117 0.675 0.608 0.508 0.458 0.317 0.383 0.375 0.358
      fe_25 fe_26 fe_27 fe_28 fe_29
fe_2
fe_3
fe_4
fe_5
fe_6
fe_7
fe_8
fe_9
fe_10
fe_11
```

```
fe_12
fe_13
fe_14
fe_15
fe_16
fe_17
fe_18
fe_19
fe_20
fe_21
fe_22
fe_23
fe_24
fe_25
fe_26 0.525
fe_27 0.492 0.200
fe_28 0.567 0.208 0.242
fe_29 0.492 0.033 0.167 0.242
fe_30 0.608 0.250 0.283 0.042 0.283
```

Step 2 + 3 - PCoA and quality

Compute multimensional functional spaces and assess their quality

```
fspaces_quality <- mFD::quality.fspaces(
  sp_dist             = sp_dist_matrix,
  maxdim_pcoa         = 10,
  deviation_weighting = "absolute",
  fdist_scaling       = FALSE,
  fdendro             = "average")
```

```
Registered S3 method overwritten by 'dendextend':
  method     from
  rev.hclust vegan
```

```
round(fspaces_quality$"quality_fspaces", 3)      # Quality metrics of spaces and the list with otl
```

```
              mad
pcoa_1d     0.197
pcoa_2d     0.103
pcoa_3d     0.052
pcoa_4d     0.042
pcoa_5d     0.049
pcoa_6d     0.057
pcoa_7d     0.062
pcoa_8d     0.066
pcoa_9d     0.070
```

```
pcoa_10d     0.073
tree_average 0.094
```

Illustrating the quality of the selected functional spaces

This function generates a figure with three panels (in rows) for each selected functional space (in columns). Each column represents a functional space, the value of the quality metric is written on the top of each column. The x-axis of all panels represents trait-based distances. The y-axis is different for each row. Looking at panels, we can see that the 4D space is the one in which points are the closest to the 1:1 line on the top row,and the closest to the x-axis for the two bottom rows, which reflects a better quality compared to other functional spaces / dendrogram. For the dendrogram, we can see on the top row that species pairs arrange in horizontal lines, meaning that different trait-based distances have then the same cophenetic distance on the dendrogram.

plot this

```
mFD::quality.fspaces.plot(
  fspaces_quality            = fspaces_quality,
  quality_metric             = "mad",
  fspaces_plot               = c("tree_average", "pcoa_2d", "pcoa_3d",
                                 "pcoa_4d", "pcoa_5d", "pcoa_6d"),
  name_file                  = NULL,
  range_dist                 = NULL,
  range_dev                  = NULL,
  range_qdev                 = NULL,
  gradient_deviation         = c(neg = "darkblue", nul = "grey80", pos = "darkred"),
  gradient_deviation_quality = c(low = "yellow", high = "red"),
  x_lab                      = "Trait-based distance")
```

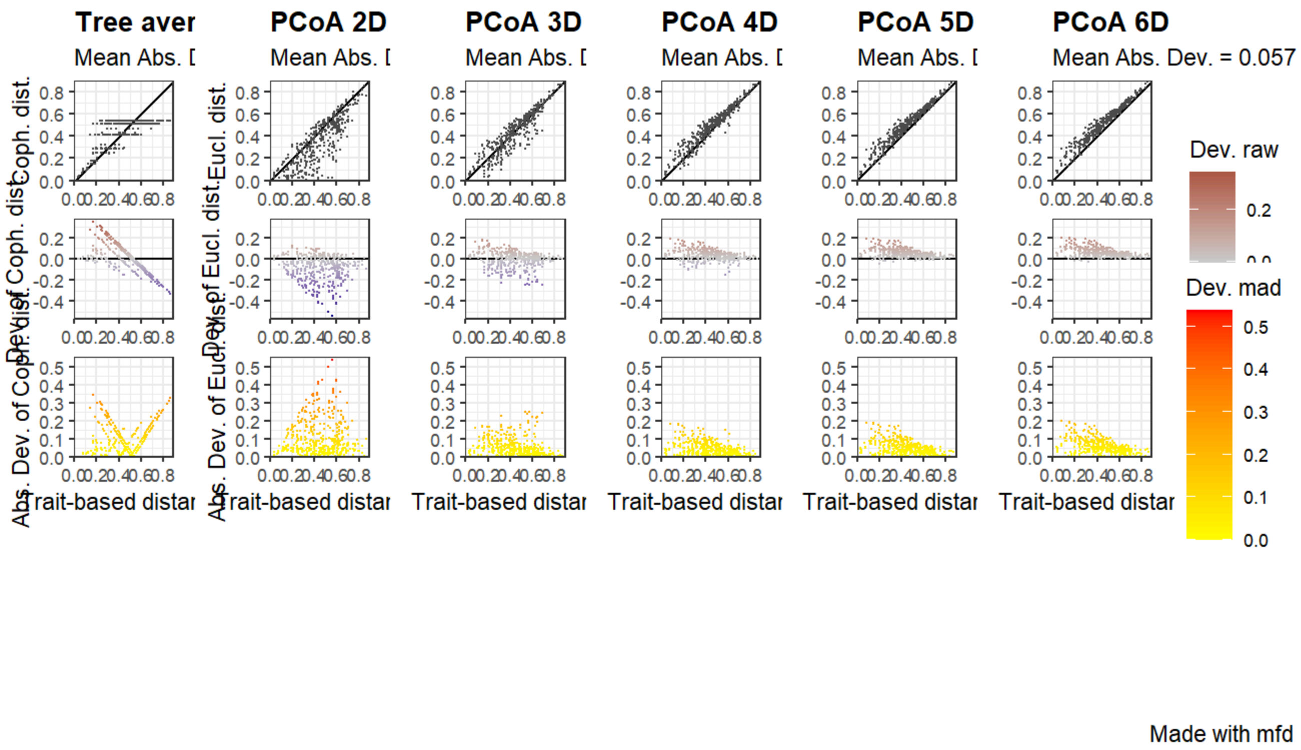

Step 4 - Correlations

```
sp_faxes_coord <- fspaces_quality$"details_fspaces"$"sp_pc_coord"

tr_faxes <- mFD::traits.faxes.cor(
  sp_tr          = species.traits.FE,
  sp_faxes_coord = sp_faxes_coord[ , c("PC1", "PC2", "PC3", "PC4")],
  plot           = TRUE)

# Print traits with significant effect:
tr_faxes$"tr_faxes_stat"[which(tr_faxes$"tr_faxes_stat"$"p.value" < 0.05), ]
```

```
               trait axis           test stat value p.value
1               Size  PC1 Kruskal-Wallis eta2 0.368  0.0167
2               Size  PC2 Kruskal-Wallis eta2 0.298  0.0328
3               Size  PC3 Kruskal-Wallis eta2 0.253  0.0499
6           Mobility  PC2 Kruskal-Wallis eta2 0.239  0.0145
7           Mobility  PC3 Kruskal-Wallis eta2 0.784  0.0000
8           Mobility  PC4 Kruskal-Wallis eta2 0.329  0.0043
9      Activity_text  PC1 Kruskal-Wallis eta2 0.437  0.0010
10     Activity_text  PC2 Kruskal-Wallis eta2 0.605  0.0001
13         Schooling  PC1 Kruskal-Wallis eta2 0.604  0.0008
```

```
17           Position  PC1 Kruskal-Wallis eta2 0.648  0.0001
21 Diet_Mouillot_text  PC1 Kruskal-Wallis eta2 0.397  0.0075
22 Diet_Mouillot_text  PC2 Kruskal-Wallis eta2 0.602  0.0008
24 Diet_Mouillot_text  PC4 Kruskal-Wallis eta2 0.614  0.0007
```

```
# Return plots:
tr_faxes$"tr_faxes_plot"
```

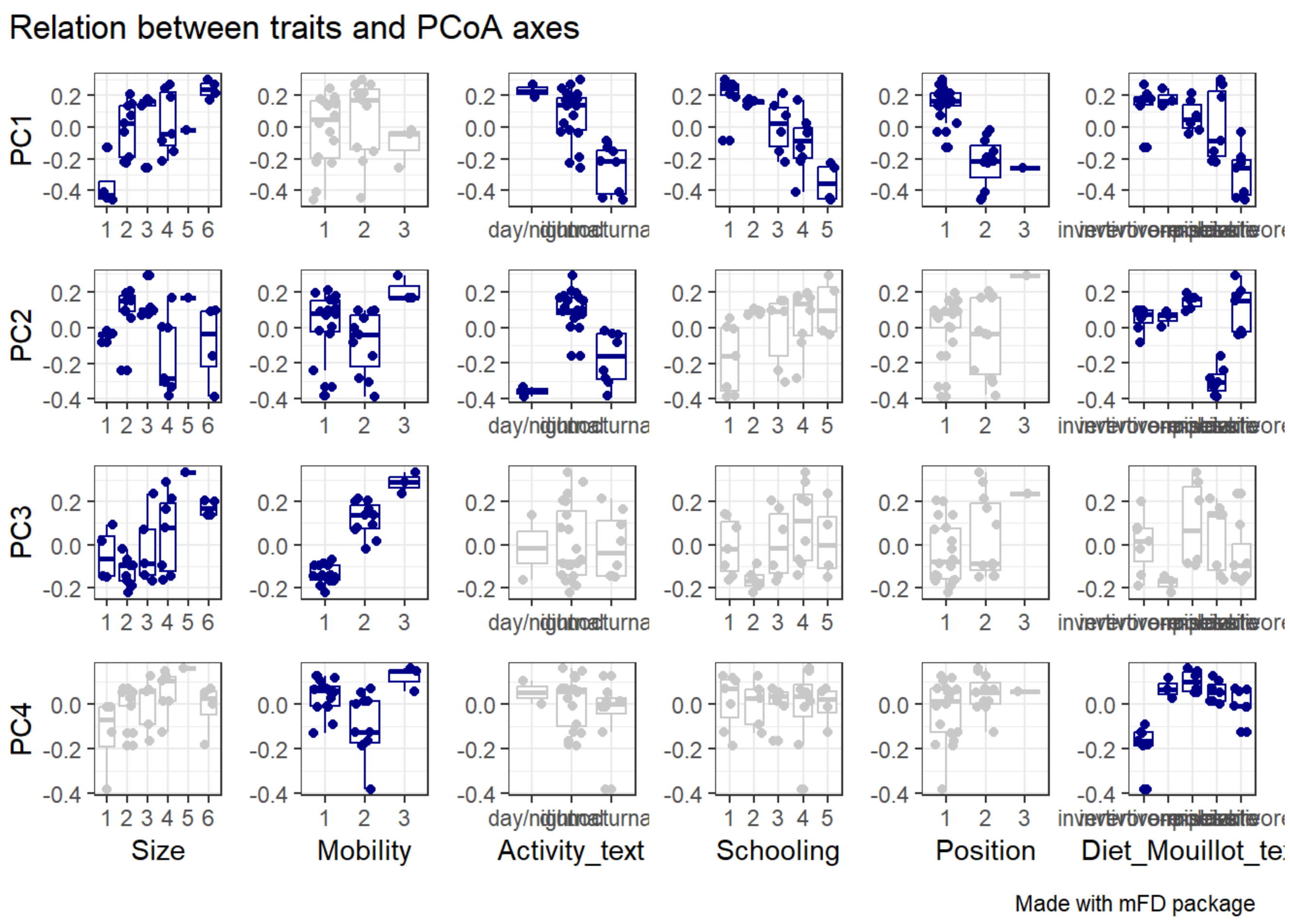

Step 5 - Plot functional space

Functional alpha diversity indices in a multidimensional space

```
#BINOMIAL to FE mapping

sp_fe <- as.data.frame(sp_to_fe$"sp_fe") # get the fe names for each species
sp_fe <- sp_fe %>% rownames_to_column() %>%
  rename(FE = `sp_to_fe$sp_fe`,
         BINOMIAL = rowname)

species.assemblage.FE <- data %>% select(U_ID, BINOMIAL, MAX_N)

species.assemblage.FE<-left_join(species.assemblage.FE, sp_fe, by ="BINOMIAL") %>% select(-BINOMI
```

```
# Pivot to wide format
species.assemblage.FE <- species.assemblage.FE %>%  pivot_wider(names_from = FE,
                                       values_from = MAX_N,
                                       values_fn = list(MAX_N = sum),
                                       values_fill = 0)

# U_ID must be the rowname
species.assemblage.FE <- species.assemblage.FE %>% column_to_rownames(var="U_ID")
```

```
# BINOMIAL to FE mapping
species.assemblage.FE$sum_FE <- rowSums(species.assemblage.FE>0)

species.assemblage.FE <- species.assemblage.FE %>%
  filter(sum_FE >= 3) %>% select(-sum_FE)
```

```
alpha_fd_indices <- mFD::alpha.fd.multidim(
  sp_faxes_coord   = sp_faxes_coord[ , c("PC1", "PC2")],
  asb_sp_w         = as.matrix(species.assemblage.FE),
  ind_vect         = c("fdis", "fmpd", "fnnd", "feve", "fric", "fdiv", "fori",
                       "fspe", "fide"),
  scaling          = TRUE,
  check_input      = TRUE,
  details_returned = TRUE)
```

```
Warning in check.asb.sp.w(asb_sp_w): Some species are absent from all
assemblages.
Warning in check.asb.sp.w(asb_sp_w): Some species are absent from all
assemblages.

Yongala_1_Antipatharia done 4%

Yongala_1_Scleractinia done 8%

Yongala_2_Antipatharia done 12%

Yongala_3_Antipatharia done 16%

Yongala_3_Scleractinia done 20%

Yongala_5_Scleractinia done 24%

Yongala_6_Antipatharia done 28%

Yongala_6_Scleractinia done 32%

Yongala_7_Scleractinia done 36%
```

```
Yongala_8_Scleractinia done 40%

Yongala_9_Antipatharia done 44%

Yongala_10_Antipatharia done 48%

Yongala_10_Scleractinia done 52%

Yongala_11_Antipatharia done 56%

Yongala_12_Antipatharia done 60%

Yongala_13_Antipatharia done 64%

Yongala_14_Scleractinia done 68%

Yongala_15_Antipatharia done 72%

Yongala_17_Antipatharia done 76%

Orpheus_18_Antipatharia done 80%

Orpheus_18_Scleractinia done 84%

Orpheus_19_Scleractinia done 88%

Orpheus_21_Antipatharia done 92%

Orpheus_22_Scleractinia done 96%

Orpheus_23_Antipatharia done 100%
```

A data frame gathering indices values in each colonies - for FIde values, there are as many columns as there are axes to the studied functional space).in our case just 2.

```
fd_ind_values <- alpha_fd_indices$"functional_diversity_indices"
fd_ind_values
```

```
                        sp_richn       fdis      fmpd      fnnd     feve
Yongala_1_Antipatharia         4 0.15637474 0.4695799 0.2362751 0.354580
Yongala_1_Scleractinia         3 0.07038737 0.2182253 0.3151698 0.408047
Yongala_2_Antipatharia         5 0.13015001 0.4385494 0.2657531 0.221874
Yongala_3_Antipatharia         5 0.22214191 0.5819370 0.2663772 0.397386
Yongala_3_Scleractinia         5 0.12097193 0.6088003 0.3452514 0.355727
Yongala_5_Scleractinia         3 0.25365310 0.9820988 2.1385431 0.220075
Yongala_6_Antipatharia         4 0.30003843 0.7126453 0.5036459 0.369016
Yongala_6_Scleractinia         6 0.54846557 0.6888040 0.7193344 0.468584
Yongala_7_Scleractinia         3 0.10987451 0.2327453 0.3486433 0.352009
Yongala_8_Scleractinia         3 0.25450555 0.4384488 0.9146473 0.597663
Yongala_9_Antipatharia         6 0.18834983 0.7166607 1.6667980 0.689596
Yongala_10_Antipatharia        5 0.11871420 0.8499042 1.6901568 0.264616
```

Yongala_10_Scleractinia	3	0.29526026	0.6849644	1.6210955	0.657162
Yongala_11_Antipatharia	6	0.21022147	0.6278312	0.1565853	0.197859
Yongala_12_Antipatharia	5	0.07779649	0.8706841	1.6989911	0.178607
Yongala_13_Antipatharia	4	0.04300817	0.8122687	1.7767318	0.060380
Yongala_14_Scleractinia	3	0.35875074	1.0412482	1.8806057	0.146411
Yongala_15_Antipatharia	4	0.06794314	0.7812007	1.7170043	0.046675
Yongala_17_Antipatharia	6	0.13082151	0.6984909	0.3611399	0.254975
Orpheus_18_Antipatharia	8	0.49898897	0.7243228	1.1177517	0.460333
Orpheus_18_Scleractinia	4	0.20911388	0.9234854	2.0406043	0.522826
Orpheus_19_Scleractinia	3	0.08664824	0.6890732	1.9643617	0.536943
Orpheus_21_Antipatharia	5	0.39999131	0.7879282	1.7214963	0.303581
Orpheus_22_Scleractinia	4	0.56915931	0.7251467	1.5623552	0.875390
Orpheus_23_Antipatharia	6	0.55183477	0.8053459	2.0986459	0.812849

	fric	fdiv	fori	fspe	fide_PC1
Yongala_1_Antipatharia	0.011054310	0.8681616	0.2355775	0.5595842	-0.1659111
Yongala_1_Scleractinia	0.005787516	0.4916140	0.2435593	0.5488667	-0.1822484
Yongala_2_Antipatharia	0.023619358	0.9534424	0.2380459	0.5655594	-0.1745444
Yongala_3_Antipatharia	0.043178418	0.9261278	0.2335760	0.5689400	-0.1524519
Yongala_3_Scleractinia	0.271123989	0.9729772	0.2582342	0.5626897	-0.1726606
Yongala_5_Scleractinia	0.220924427	0.9340806	0.2504606	0.6339800	-0.1755247
Yongala_6_Antipatharia	0.213601438	0.9740060	0.2734256	0.6295456	-0.1656719
Yongala_6_Scleractinia	0.393637635	0.8106993	0.2199447	0.6487626	-0.1836647
Yongala_7_Scleractinia	0.005787516	0.6277558	0.2361126	0.5544059	-0.1800316
Yongala_8_Scleractinia	0.006185917	0.6825587	0.1907155	0.4639100	-0.1004963
Yongala_9_Antipatharia	0.342564465	0.9545579	0.1993030	0.8947076	-0.4076827
Yongala_10_Antipatharia	0.167016334	0.9454278	0.1375755	0.9314360	-0.4279286
Yongala_10_Scleractinia	0.059895658	0.8583107	0.1531077	0.8572932	-0.3830899
Yongala_11_Antipatharia	0.174955510	0.8907592	0.1463457	0.9111221	-0.4140499
Yongala_12_Antipatharia	0.165269664	0.9840819	0.1343619	0.9635420	-0.4464397
Yongala_13_Antipatharia	0.429412518	0.9501202	0.1318377	0.9721506	-0.4523904
Yongala_14_Scleractinia	0.326068105	0.7204493	0.2761478	0.7033941	-0.1978165
Yongala_15_Antipatharia	0.157775037	0.9773902	0.1337750	0.9660202	-0.4483138
Yongala_17_Antipatharia	0.562647862	0.9030762	0.1953081	0.9004328	-0.4065201
Orpheus_18_Antipatharia	0.429091001	0.8218788	0.2325943	0.6415857	-0.1895402
Orpheus_18_Scleractinia	0.384774079	0.9803676	0.2638625	0.6508394	-0.1980545
Orpheus_19_Scleractinia	0.003965864	0.9825934	0.2453867	0.6356335	-0.2073157
Orpheus_21_Antipatharia	0.229681042	0.8969438	0.2362630	0.6102365	-0.1319579
Orpheus_22_Scleractinia	0.272191901	0.8421433	0.2255332	0.5806704	-0.1240635
Orpheus_23_Antipatharia	0.260136256	0.9826490	0.2309260	0.6524535	-0.1945368

	fide_PC2
Yongala_1_Antipatharia	0.175103119
Yongala_1_Scleractinia	0.178221245
Yongala_2_Antipatharia	0.179582086
Yongala_3_Antipatharia	0.170083923
Yongala_3_Scleractinia	0.164235339
Yongala_5_Scleractinia	0.172485446
Yongala_6_Antipatharia	0.143130934
Yongala_6_Scleractinia	0.042383256
Yongala_7_Scleractinia	0.182193621
Yongala_8_Scleractinia	0.156552850
Yongala_9_Antipatharia	-0.025848244

```
Yongala_10_Antipatharia -0.021054631
Yongala_10_Scleractinia  0.009966482
Yongala_11_Antipatharia  0.004955137
Yongala_12_Antipatharia -0.007235361
Yongala_13_Antipatharia -0.013809207
Yongala_14_Scleractinia  0.130212801
Yongala_15_Antipatharia -0.008546718
Yongala_17_Antipatharia -0.031075222
Orpheus_18_Antipatharia  0.062182999
Orpheus_18_Scleractinia  0.169139859
Orpheus_19_Scleractinia  0.199857902
Orpheus_21_Antipatharia  0.150534871
Orpheus_22_Scleractinia  0.038899908
Orpheus_23_Antipatharia -0.009448386
```

```
details_list <- alpha_fd_indices$"details"
```

Extract the functional richness space coordinates for each colony

```
# Global functional richness space - all sites all colonies
global.cords<- as.data.frame(details_list[["asb_G_coord"]])  # Extract by name if list has named 
global.cords<- global.cords %>% rownames_to_column("axes")

global.cords <- global.cords %>%
  pivot_longer(-axes, names_to = "U_ID", values_to = "Value") %>%
  pivot_wider(names_from = axes, values_from = Value)

global.cords <- global.cords %>% mutate(
  SITE = case_when(str_detect(U_ID, "Yongala") ~ "Yongala",
  TRUE ~ "Orpheus"),
  ORDER = case_when(str_detect(U_ID, "Anti") ~ "Antipatharia",
  TRUE ~ "Scleractinia"),
  GLOBAL = "Global"
)
```

Functional trait space plot with convex hull

```
hulls <- global.cords %>% group_by(ORDER) %>%
slice(chull(PC1, PC2))

global.hull <-global.cords %>% select(-ORDER) %>%  group_by(SITE) %>%
slice(chull(PC1, PC2))

functional.space.plot.1<-ggplot(global.cords, aes( x = PC1, y =PC2, colour = ORDER)) +
```

```
  geom_polygon(data = hulls, alpha = 0.4,
               aes(fill = ORDER,colour = ORDER, linetype = ORDER)) +
   geom_polygon(data = global.hull, alpha = 0,
               aes(fill = GLOBAL,colour = GLOBAL,linetype = GLOBAL)) +
  scale_fill_manual(values = c("#EFC165","grey","#65BAC4")) +
  scale_colour_manual(values = c("#EFC165","grey","#65BAC4")) +
  scale_linetype_manual(values = c(1,3,1)) +
  theme_bw() +
  theme(
    legend.title = element_blank(),
    legend.position = "bottom",
  )

functional.space.plot.1
```

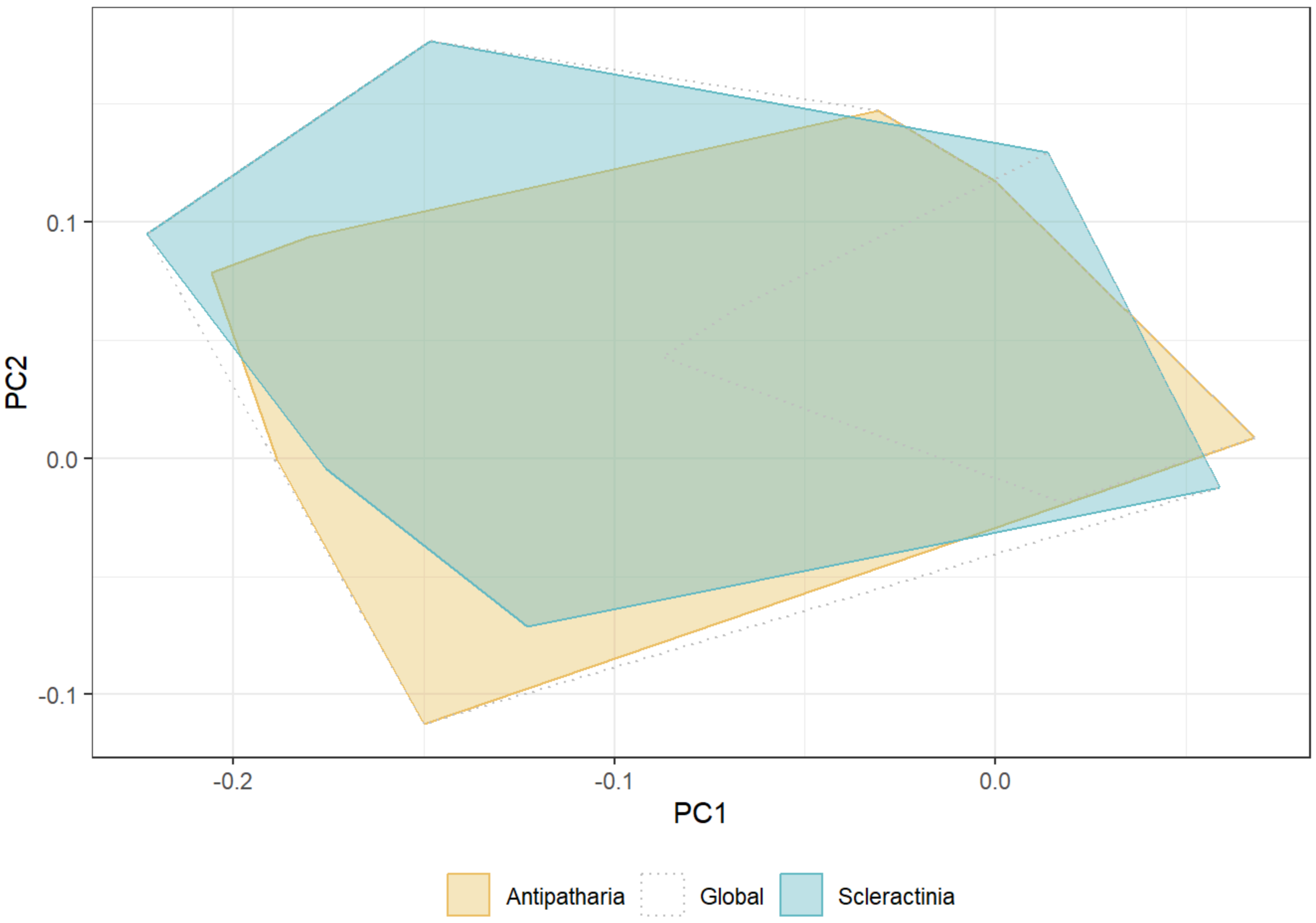

Table 1

final_diversity_summary

U_ID	richness_taxonomic	diversity_shannon	diversity_simpson	nb_sp	nb_fe	fred
**Yongala_1_Antipatharia**	5	1.106956616892020	0.5612366230677760	5	4	1.25
**Yongala_1_Scleractinia**	3	0.7595473914748640	0.4297520661157030	3	3	1
**Yongala_2_Antipatharia**	5	1.028181455698950	0.5644444444444450	5	5	1
**Yongala_2_Scleractinia**	1	0	0	1	1	1
**Yongala_3_Antipatharia**	6	1.346905192247290	0.6538271604938270	6	5	1.2
**Yongala_3_Scleractinia**	5	0.6801682153610050	0.3223363980530020	5	5	1
**Yongala_4_Antipatharia**	2	0.5982695885852570	0.40816326530612200	2	1	2
**Yongala_4_Scleractinia**	1	0	0	1	1	1
**Yongala_5_Antipatharia**	3	0.9429491606819860	0.5596707818930040	3	2	1.5
**Yongala_5_Scleractinia**	3	0.4702359623552490	0.2344045368620040	3	3	1
**Yongala_6_Antipatharia**	5	1.0561841477588400	0.5625	5	4	1.25
**Yongala_6_Scleractinia**	6	1.430280301911090	0.6933333333333330	6	6	1
**Yongala_7_Antipatharia**	2	0.21903252178902100	0.10775510204081600	2	2	1
**Yongala_7_Scleractinia**	4	1.1709971261162100	0.6428571428571430	4	3	1.3333333333333300
**Yongala_8_Antipatharia**	3	0.8090881237818760	0.49537037037037000	3	2	1.5
**Yongala_8_Scleractinia**	3	0.9369181202504690	0.5578512396694220	3	3	1
**Yongala_9_Antipatharia**	7	0.5902797223440610	0.24471055639886800	7	6	1.1666666666666700
**Yongala_9_Scleractinia**	1	0	0	1	1	1
**Yongala_10_Antipatharia**	5	0.3498029175594710	0.14643046799471700	5	5	1
**Yongala_10_Scleractinia**	3	0.6441798059733930	0.3698224852071010	3	3	1
**Yongala_11_Antipatharia**	6	1.1454661414326500	0.622001836547291	6	6	1
**Yongala_11_Scleractinia**	2	0.6931471805599450	0.5	2	2	1
**Yongala_12_Antipatharia**	6	0.257589212901637	0.09554135523138970	6	5	1.2
**Yongala_12_Scleractinia**	3	0.28630516241243900	0.1243776749061050	3	2	1.5
**Yongala_13_Antipatharia**	4	0.14669313147992000	0.05332404729821810	4	4	1
**Yongala_13_Scleractinia**	2	0.5779217946828970	0.38927335640138400	2	2	1
**Yongala_14_Antipatharia**	1	0	0	1	1	1
**Yongala_14_Scleractinia**	3	0.6001660731596460	0.31404958677685900	3	3	1
**Yongala_15_Antipatharia**	4	0.20152720711603400	0.08295368695771620	4	4	1
**Yongala_15_Scleractinia**	1	0	0	1	1	1
**Yongala_16_Antipatharia**	3	0.7338321983300770	0.453254132231405	3	2	1.5
**Yongala_16_Scleractinia**	1	0	0	1	1	1
**Yongala_17_Antipatharia**	7	1.0302833906079500	0.5752335975712600	7	6	1.1666666666666700
**Yongala_17_Scleractinia**	1	0	0	1	1	1
**Orpheus_18_Antipatharia**	8	1.0732233628778100	0.5544987795640100	8	8	1
**Orpheus_18_Scleractinia**	5	0.7233627993370300	0.3366285119667020	5	4	1.25
**Orpheus_19_Antipatharia**	2	0.21028341228613300	0.1022644265887510	2	2	1
**Orpheus_19_Scleractinia**	4	0.8898548823965990	0.5451843043995240	4	3	1.3333333333333300
**Orpheus_20_Antipatharia**	2	0.2337916587064590	0.1171875	2	2	1
**Orpheus_20_Scleractinia**	1	0	0	1	1	1
**Orpheus_21_Antipatharia**	5	0.8740735854074160	0.40955004591368200	5	5	1
**Orpheus_21_Scleractinia**	3	0.8033149761408760	0.48	3	2	1.5
[illegible]	[illegible]	[illegible]	[illegible]	[illegible]	[illegible]	[illegible]

**Orpheus_22_Antipatharia**	2	0.10473243910508700	0.04253308128544430	2	2	1
**Orpheus_22_Scleractinia**	4	1.3229495110045300	0.72	4	4	1
**Orpheus_23_Antipatharia**	7	1.1493550641126400	0.608196159122085	7	6	1.1666666666666700
**Orpheus_23_Scleractinia**	3	0.9832739442463890	0.6015625	3	2	1.5
